# Unveiling the potent effect of vitamin D: harnessing Nrf2/HO-1 signaling pathways as molecular targets to alleviate urban particulate matter-induced asthma inflammation

**DOI:** 10.1186/s12890-024-02869-2

**Published:** 2024-01-25

**Authors:** Dandan Ge, Qihong Chen, Xiaohua Xie, Qiyuan Li, Yungang Yang

**Affiliations:** 1 Department of Pediatrics, Pediatric Key Laboratory of Xiamen, Institute of Pediatrics, School of Medicine, The First Affiliated Hospital of Xiamen University, Xiamen University, Zhenhai Road No.55, Xiamen, 361003 China; 2 National Institute for Data Science in Health and Medicine, School of Medicine, Xiamen University, Xiang’an South Road, Xiamen, 361102 China

**Keywords:** Vitamin D, Asthma, Particulate matters, HO-1, Nerve growth factor

## Abstract

**Background:**

Asthma is the most common allergic disease characterized by an inflammatory response in the airways. Mechanismly, urban particulate matter (PM) is the most widely air pollutant associated with increased asthma morbidity and airway inflammation. Current research found that vitamin D is an essential vitamin with anti-inflammatory, antioxidant and other medical efficacy. Inadequate or deficient vitamin D often leads to the pathogenesis and stability of asthma. NGF exacerbates airway inflammation in asthma by promoting smooth muscle cell proliferation and inducing the Th2 immune response. Activation of the Nrf2/HO-1 signaling pathway can exert a protective effect on the inflammatory response in bronchial asthma. However, the specific mechanism of this pathway in PM-involved asthmatic airway smooth muscle cells remains unclear.

**Methods:**

Mice were sensitized and challenged with Ovalbumin (OVA) to establish an asthma model. They were then exposed to either PM, vitamin D or a combination of both, and inflammatory responses were observed. Including, acetylcholine stimulation at different concentrations measured airway hyperresponsiveness in mice. Bronchoalveolar lavage fluid (BALF) and serum were collected for TNF-α, IL-1β, IL-6, and Nerve growth factor (NGF) analysis. Additionally, lung tissues underwent histopathological examination to observe alveolar structure and inflammatory cell infiltration. Specific ELISA kits were utilized to determine the levels of the inflammatory factors TNF-α, IL-1β, IL-6, and Nerve growth factor (NGF). Nrf2/HO-1 signaling pathways were examined by western blot analysis. Meanwhile, we constructed a cell system with low HO-1 expression by lentiviral transfection of airway smooth muscle cells. The changes of Nrf2, HO-1, and NGF were observed after the treatment of OVA, PM, and Vit D were given.

**Results:**

The in vivo results showed that vitamin D significantly alleviated pathological changes in lung tissue of PM-exposed mice models. Mechanismly, vitamin D decreased substantial inflammatory cell infiltration in lung tissue, as well as the number of inflammatory cells in BALF. Furthermore, vitamin D reduced the heightened inflammatory factors including of TNF-α, IL-1β, IL-6, and NGF caused by PM exposure, and triggered the activity of nucleus Nrf2 and HO-1 in PM-exposed asthmatic mice. Notably, knockdown HO-1 weakens the Vitamin D- mediated inhibition to pollution toxicity in asthma. Importantly, in vitro experiments on OVA-stimulated mice airway smooth muscle cells, the results showed that OVA and PM, respectively, reduced Nrf2/HO-1 and increased NGF’s expression, while vitamin D reversed the process. And in the HO-1 knockdown cell line of Lenti-si-HO-1 ASMCs, OVA and PM reduced Nrf2’s expression, while HO-1 and NGF’s expression were unchanged.

**Conclusions:**

The above results demastrate that vitamin D downregulated the inflammatory response and the expression of NGF by regulating the Nrf2/HO-1 signaling pathways in airway smooth muscle cells, thereby showing potent anti-inflammatory activity in asthma.

**Supplementary Information:**

The online version contains supplementary material available at 10.1186/s12890-024-02869-2.

## Background

Asthma is a chronic inflammatory airways disease with marked heterogeneity and complex pathophysiological manifestations. Currently, there are at least 300 million asthma cases worldwide, and the prevalence is increasing yearly, with an additional one-third of patients expected by 2025 [1]. Asthma is mainly caused by genetic susceptibility and environmental exposure, with a growing number of cases attributed to airborne particles each year. Of note, the International Agency for Research on Cancer (IARC) has ranked airborne particulate matter (PM) as a class I carcinogen [2]. In addition, many studies have demonstrated that asthma cases are exacerbated by geographic exposure to airborne particulate matter [3–6].

Vitamin D3 is a fat-soluble vitamin with various pharmaceutical properties, including anti-inflammatory, immunomodulatory activities, the promotion of barrier function, wound healing and hair growth, and anticancer [7, 8]. Vitamin D supplementation modulates the host immune response to viruses [9]. Vitamin D levels in peripheral blood are low in patients with systemic sclerosis [10]. Importantly, vitamin D deficiency has been linked to the increase in asthma incidence over the past 20 years [11–13]. Extensive epidemiological studies have shown that vitamin D supplementation can enhance the antiviral response of respiratory epithelial cells [14] and inhibit IL-17 A secretion in the peripheral blood of severe asthma patients [15, 16]. Additionally, low concentrations of vitamin D metabolite 1α, 25-hydroxyvitamin D3, were associated with an increased risk of childhood acute asthma exacerbations [17]. Of note, Jolliffe reported that vitamin D supplementation significantly reduced the incidence of acute exacerbations in asthma patients treated with systemic corticosteroids [18]. A study in the United States showed that vitamin D-deficient children living near major roads, high in PM exposure, were five times more likely to develop asthma than those who were vitamin D-sufficient, and living in the same areas [19]. This study demonstrated that vitamin D has antioxidant effects and that traffic-related air pollution contributed to asthma morbidity. Vitamin D deficiency exacerbates airway oxidative stress induced by air pollutants, leading to an increase in severe asthma exacerbations in children.

Multiple signaling pathways are known to be involved in the pathophysiology of asthma. For example, Nrf2 has been reported to be a crucial redox-sensitive transcription factor by redox homeostasis cellular maintenance through the regulation of numerous downstream antioxidants such as heme oxygenase-1 (HO-1), NAD(P)H dehydrogenase quinone 1 (NQO1), and superoxide dismutase (SOD) [20]. Of note, HO-1 is a rate-limiting enzyme in heme degradation [21], that regulates redox reactions and has cytoprotective effects against oxidative stress-induced cellular damage. HO-1’s expression is induced in the airways of asthmatic patients and has been shown to slow down allergic airway inflammation [22]. Specifically, the induction of HO-1 expression reduces the production of reactive oxygen species (ROS) in the airways and alleviates IL-13-induced goblet cell proliferation, finally preventing asthma development [23]. Therefore, targeting the Nrf2/HO-1 signaling pathway may be an effective strategy for asthma prevention.

It has been shown that vitamin D prevents the development of asthma linked to traffic-related air pollution exposure. Specifically, an earlier study has shown that prenatal and postnatal vitamin D supplementation significantly attenuated lung inflammation and differentiation of Th2 in asthma linked to PM exposure [12]. This data suggests that vitamin D supplementation reduces children’s susceptibility to the adverse effects of PM. However, the molecular mechanisms of vitamin D in PM-exposed asthma are unclear. In this study, we aimed to evaluate the effects of vitamin D on OVA-induced allergic asthma in PM-exposed mice and further investigate its underlying molecular mechanisms in vitro and in vivo.

## Methods

### Reagents and antibodies

The Ovalbumin (OVA), Aluminum hydroxide, and TEMED were purchased from Sigma-Aldrich. The Eastep™ Total RNA Extraction Kit and GoTaq® qPCR Master Mix kit were obtained from Promega. The Strong RIPA lysate was purchased from Boster. The protein phosphatase inhibitor complex and protease inhibitors were purchased from Roche. The BCA protein quantitative kit, cDNA synthesis kit, and TRIzol Total RNA Extraction Kit were purchased from Tiangen. The ECL chemiluminescence detection kit was purchased from Advansta. The Multicolor Prestained Protein Ladder was purchased from Thermo Fisher Scientific. The PVDF membrane was purchased from EMD Millipore (Billerica, MA). The 4×loading buffer (containing DTT) was purchased from Solarbio. The Cell Proliferation Assay (CellTiter 96® AQ_ueous_ One Solution, Cat. G3582) was purchased from Promega. The Super-PAGE™ Bis-Tris Gels and nucleoprotein extraction kit were purchased from Shanghai Epizyme Biomedical Technology. Recombinant mouse TNFα (Cat. 50,349-MNAE) protein was purchased from Sino Biological.

The Nrf2 rabbit pAb (Cat. NBP1-32822) was purchased from Novus Biologicals. HO-1 mouse mAb (Cat. SMC-131D-P594) was purchased from StressMarq Biosciences, and the Histone H3 (Cat. 4620 S) rabbit mAb were purchased from Cell Signaling Technology. The Mouse β-Actin (Cat. MAB8929) antibody was purchased from R&D systems. The mouse iNOS (Cat.ab178945) antibody was pruchased from Abcam. The Goat Anti-Rabbit IgG (Cat. BA1054) and Goat Anti-Mouse IgG (Cat. BA1050) were purchased from Boster. The mouse TNFα (Cat. VAL609), IL-1β(Cat. VAL601), IL-6 (Cat. VAL604) ELISA Valukine Kit were purchased from Novus Biologicals, and mouse NGF ELISA Kit (Cat. EK15726-1) was purchased from SAB Signalway Antibody. Urban Particulate Matter (PM) (Cat. SMR1864a) was purchased from National Institute of Standards and Technology in the United States of America and used as the reference material. 1α,25-dihydroxyvitamin D3 (Vitamin D, Cat. D1530) was purchased from Sigma-Aldrich. Oligo nucleotides were synthesized by Sangon Biotech (Shanghai, China), and all the primer sets were listed in supplementary data (Supplement table S1).

### Animal and mouse model

**Fig. 1:**
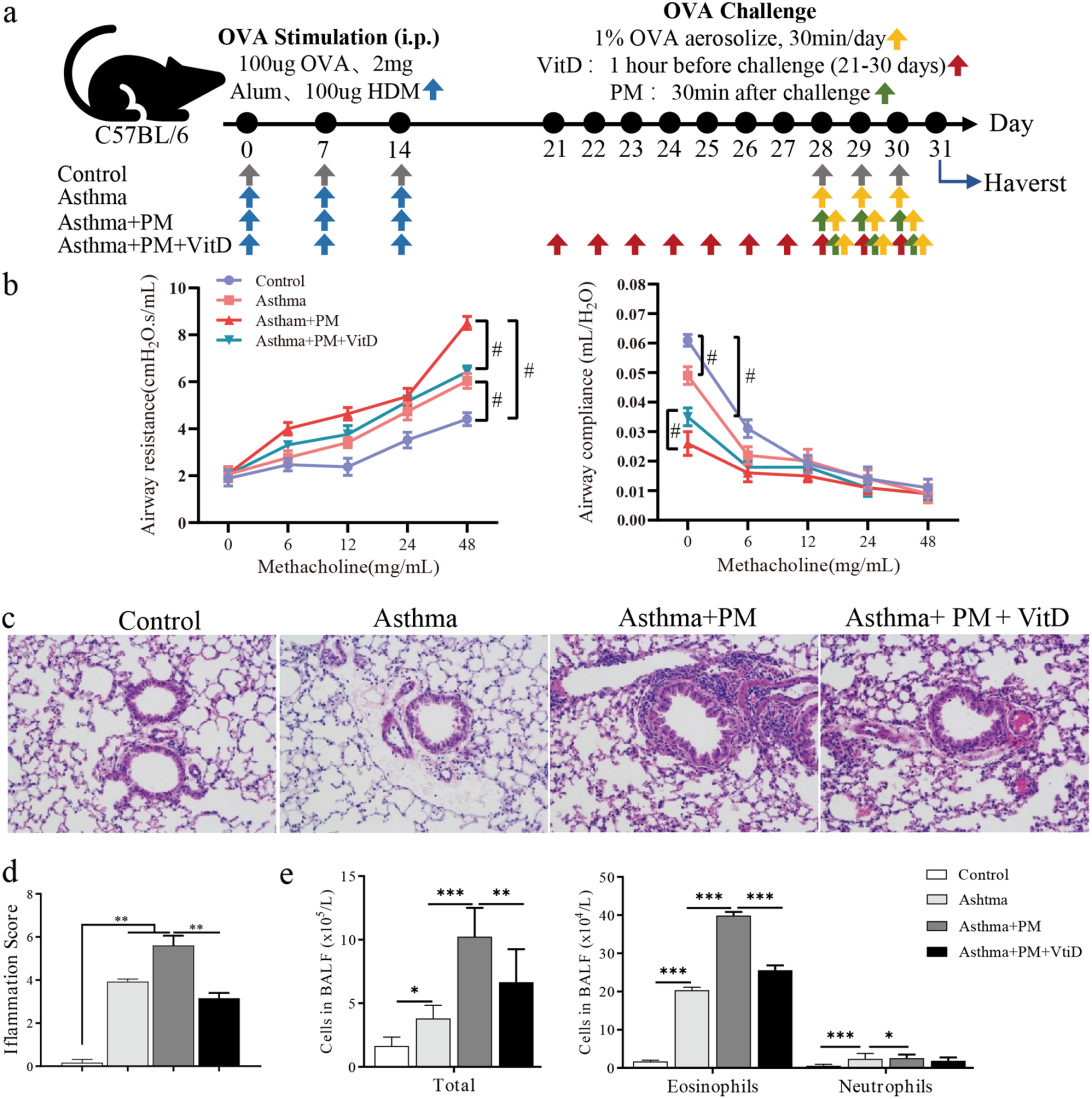
Vitamin D improves PM-exposed lung histopathological inflammation in asthmatic mice. Thirty-two female mice were randomly assigned to the Control group, Asthma group, Asthma + PM group, or Asthma + PM + Vit D group. (**a**) Schematic illustration of the mouse modeling process. The gray arrows indicate that each mouse was administered normal saline instead. The blue arrow indicates that each mouse was injected intraperitoneally with OVA sensitization solution. The yellow arrow indicates that the mice were exposed to 1% OVA for 30 min. The green arrow indicates that the mouse was intratracheally instilled with 100ug of PM. The red arrow indicates that the mouse was gavaged with Vit D. (**b**) Airways resistance (Rrs) and compliance (Crs) of different groups (^#^*p* < 0.01). (**c**) Airway inflammation (H&E staining, ×200). (**d**) Inflammatory index. (**e**) proportion of inflammatory cells in BALF (* *p* < 0.05, ** *p* < 0.01, *** *p* < 0.001, and **** *p* < 0.0001)

Wild-type BALB/c mice (female, 6–8 weeks) used in this study were obtained from the Xiamen University Laboratory Animal Center (XMULAC). All mice were maintained under specific pathogen-free (SPF) conditions at the XMULAC. Experiments on mice were approved by the Institutional Animal Care and Use Committee, and were in strict accordance with good animal practice as defined by the XMULAC. Mice were randomly divided into four groups, the control group (Control), the asthma group (Asthma), the PM-treatment asthma group (Asthma + PM), and the vitamin D and PM-treatment asthma group (Asthma + PM + Vit D). The asthma modeling and the treatment with the particulate matter were established based on earlier reports [24, 25], and adapted in line with our experiments. On days 0, 7, 14, and 21 mice received an intraperitoneal (i.p.) injection with 100 µl of sensitization liquid (100 µg OVA and 2 mg aluminum hydroxide were dispersed in 100 µl of normal saline). On days 28, 29, and 30 mice were challenged with 1% OVA stimulated by nebulization. The PM-treatment and the vitamin D and PM-treatment asthma groups were administered intranasally 20 µl PM solution (100 µg PM dissolved in 20 µl saline solution) 30 min prior to being challenged. In contrast, the asthma group was given 40 µl of normal saline. The vitamin D and PM-treatment asthma group was given 300 µl of vitamin D solution (100 ng vitamin D dissolved in ethanol, and further diluted to 0.9% by 300 µl PBS) [26] by gavage on 21–30 days. All the mice were anesthetized through the nose with isoflurane before dripping. Meanwhile, the control group was administered normal saline. Mice were sacrificed by euthanized 24 h after the last nasal drip (Fig. 1a).

### Lung function measurement

The mice airway reponsiveness was assessed at 24 h after the last OVA challenge using the FlexiVent system (SCIREQ Inc., Montreal, QC, Canada). After mice were anaesthetised with 60 mg/kg pentobarbital sodium (Sigma-Aldrich), they were tracheotomised and endotracheally intubated. Airway responsiveness was then assessed by measuring changes in pulmonary resistance after intubation. Changes in lung resistance were assessed after continuous exposure to increasing doses of methacholine (Sigma-Aldrich) (0, 6, 12, 24 and 48 mg/ml) in a sterile incubator. Airway resistance (Rrs) and compliance (Crs) values were recorded to analyse airway hyperresponsiveness (AHR).

### Flow cytometry of lung tissue

We sacrifice the mice at 24 h after the last OVA challenge. Then we harvest the lung tissue and collect the lymphocyte through collagenase digesting, grind, and 70%/42% percoll separation. Then 1ug/ml Golgi Stop was used for blocking cytokine secretion. After 5 h, we collected the cells for surface markers FACS staining at a ratio of 1:200 (anti-CD4, anti-CD8) for 15 min. Fix Buffer/Permeabilization Buffer with 1:3 ratio was added at 4℃ for 30 min.Finally, we stained Intracellular and intranuclear cytokine using antibodies at a ratio of 1:100 (anti-IFNg, anti-IL4, anti-CD4 and anti-CD8) for 15 min. The samples were checked with a BD LSRFortessa™ flow cytometer (BD Biosciences) and analyzed with Flow Jo software (version V10).

### Cell culture and lentivirus intervention

Mouse airway smooth muscle cells (ASMCs, Cat. No. iCell-m002, iCell Bioscience Inc.) were seeded in 24-well plates at 1 × 10^5^ cells per well and cultured with medium supplemented with 10% fetal bovine serum. Once 80% cell fusion was reached, the cells were starved for 24 h with a serum-free medium containing 0.1% FBS. The goal was to have the cells in each group simultaneously in the G0 phase. Vitamin D was added into the well at a concentration of 10^− 7^ nmol/L at 2 h before adding 100 µg/ml OVA and/or 100 ug/ml PM. Subsequently, either 100 µg/ml OVA, 100 µg/ml PM, or both were added. After 24 h, the cell supernatant was collected. The cells were washed three times and collected for subsequent experiments. The optimal vitamin D action time and concentration were shown in Supplementary material.

HO-1-targeted short hairpin RNAs were then designed and synthesized by Genechem Co. Ltd (Shanghai, China). Details about lentivirus construction and pre-test are shown in the Supplementary material. ASMCs were infected with the lentivirus expressing a HO-1 shRNA (Lenti-si-HO-1). The optimal infection efficiency of HO-1 sh-RNA was shown in Supplementary Fig. 2 and Supplementary Fig. 3.

A cell suspension with a density of 3–5 × 10^4^ cells/ml was prepared using a complete medium and seeded in 6-well plates. Cells were incubated for 16-24 h at 37 °C until the cell confluence reached 20–30%; then, the infection reagent HiTransG A and 200 µl of 1 × 108 TU/ml of adenovirus were added to the cells. After 16 h of incubation at 37 °C, the medium was replaced with a complete medium, and the culture was continued. After 72 h of infection, cells were observed for infection efficiency, and the best 1-well cells were selected for passaging. Puromycin at 2 ng/ml was added to the passages for positive screening (Fig. 4e). Importantly, Lenti-si-HO-1 ASMCs were treated as ASMC.

### Experimental statistical analysis

Statistical analysis was performed with GraphPad Prism 5. One-way analysis of variance (ANOVA) with Tukey’s multiple comparison test was performed to compare more than two test groups, as appropriate. A p-value of less than 0.05 was considered statistically significant.

## Results

### Vitamin D ameliorated PM-exposed lung histopathological inflammation in asthmatic mice

PM-treated asthmatic mice showed a significant increase in airways resistance (Rrs), and a significant decrease in airways compliance (Crs) compared to asthmatic mice treated with methacholine at a concentration of 48 mg/ml (Fig. 1b). While, the combination of vitamin D and PM treatment significantly decreased respiratory resistance and significantly increased respiratory compliance in asthmatic mice compared to PM-treated asthmatic mice treated with methacholine at a concentration of 48 mg/ml (Fig. 1a). Lung histopathological staining showed that the number of inflammatory cells infiltrating the airways in both the asthma and the PM-treated asthma groups was significantly higher than in the control group. At the same time, vitamin D treatment significantly reduced the number of inflammatory cells (Fig. 1c, d). In addition, the number of inflammatory cells in BALF in the asthma group was significantly higher than in the control group (especially eosinophils). With PM treatment, the number of inflammatory cells in BALF increased. However, the number of inflammatory cells was significantly reduced in PM-treated asthmatic mice after treated with vitamin D (Fig. 1e).

### Vitamin D attenuates lung inflammation in PM-exposed asthmatic mice

**Fig. 2:**
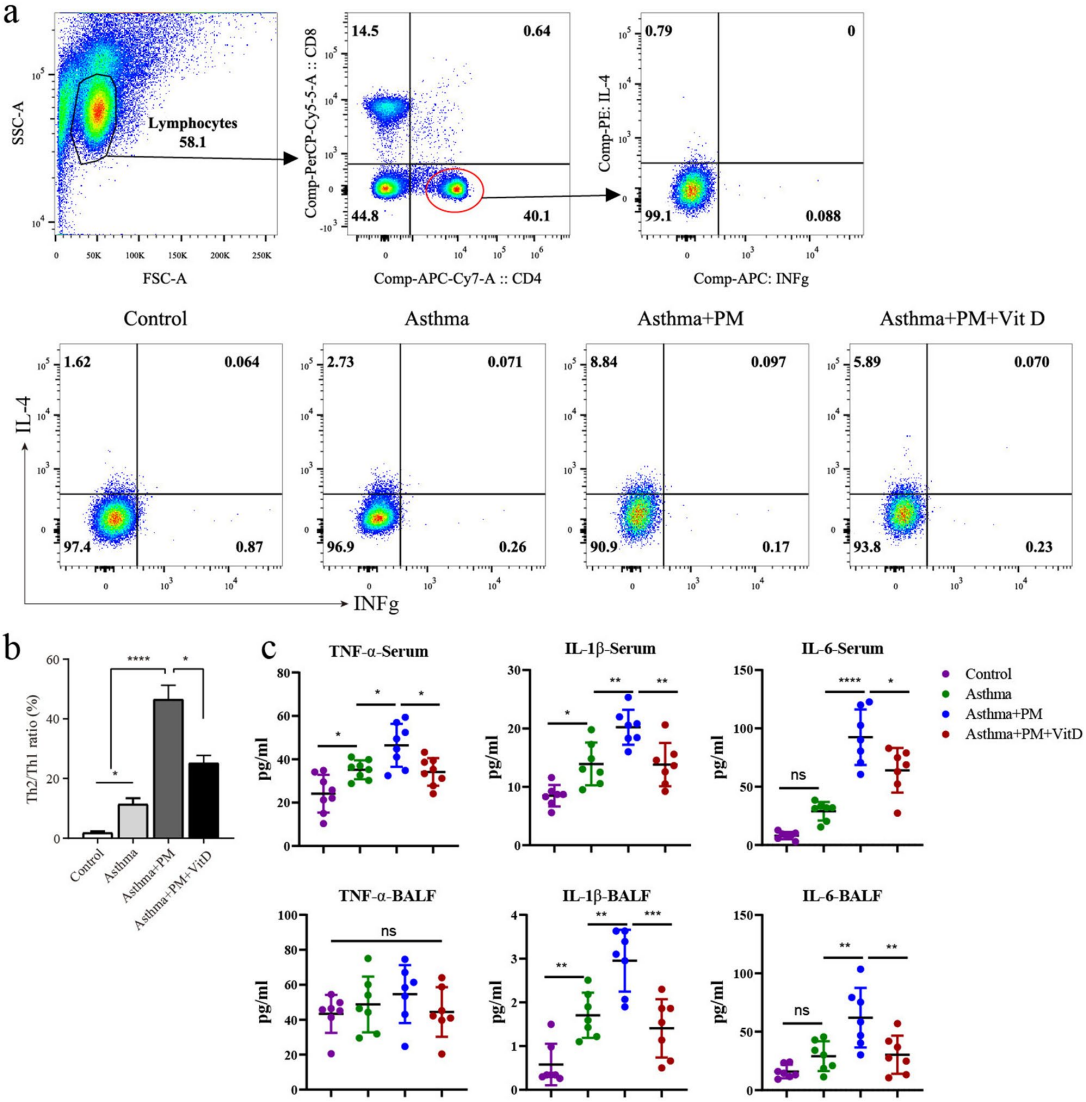
Vitamin D alleviates inflammation in the lungs of asthmatic rats. (**a**) flow cytometry analysis of Th1, Th2 cell ratios in lung tissue. (**b**) relative ratios of Th1 and Th2 cell ratios in lung tissue. (**c**) ELISA analysis of the TNFα, IL-1β and IL-6 inflammatory factor protein expression levels in BALF and serum. (* *p* < 0.05, ** *p* < 0.01, *** *p* < 0.001, and **** *p* < 0.0001)

NGF exacerbates airway inflammation in asthma by promoting smooth muscle cell proliferation and inducing the Th2 immune response [27, 28]. To further evaluate the anti-inflammatory effect of vitamin D on PM-treated asthma mice, CD4^+^ cells in lung tissue were extracted, and the ratio of cells for Th1 and Th2 was detected. Additionally, the expression levels of inflammatory factors in BALF and serum was detected. Compared with the control group, the proportion of Th2/Th1 cells in the asthma group increased, and the rise was more significant under PM treatment. However, following vitamin D administration, the proportion of Th2/Th1 cells decreased (Fig. 2a, 2b). An ELISA kit was used to detect the levels of TNF-α, IL-1β, and IL-6 in BALF neutralization and serum. The results showed that in serum, TNF-α expression in the asthma group was significantly higher than in the control group and more pronounced under PM treatment. Of note, while TNF-α expression decreased significantly following vitamin D administration, this phenomenon was not observed in BALF (Fig. 2c). In serum and BALF, IL-1β expression increased significantly in the asthma group compared with the control group. Interestingly, while the increase was more significant under PM treatment, after vitamin D administration, IL-1β expression decreased significantly (Fig. 2c). In serum and BALF, IL-6 expression in the asthma group increased but was not significant compared to the control group (*P* > 0.5). On the contrary, IL-6 expression increased significantly under PM treatment, and IL-1β expression decreased significantly after the administration of vitamin D (Fig. 2c).

### Vitamin D activates the PM-exposed Nrf2/HO-1 signaling pathway

**Fig. 3:**
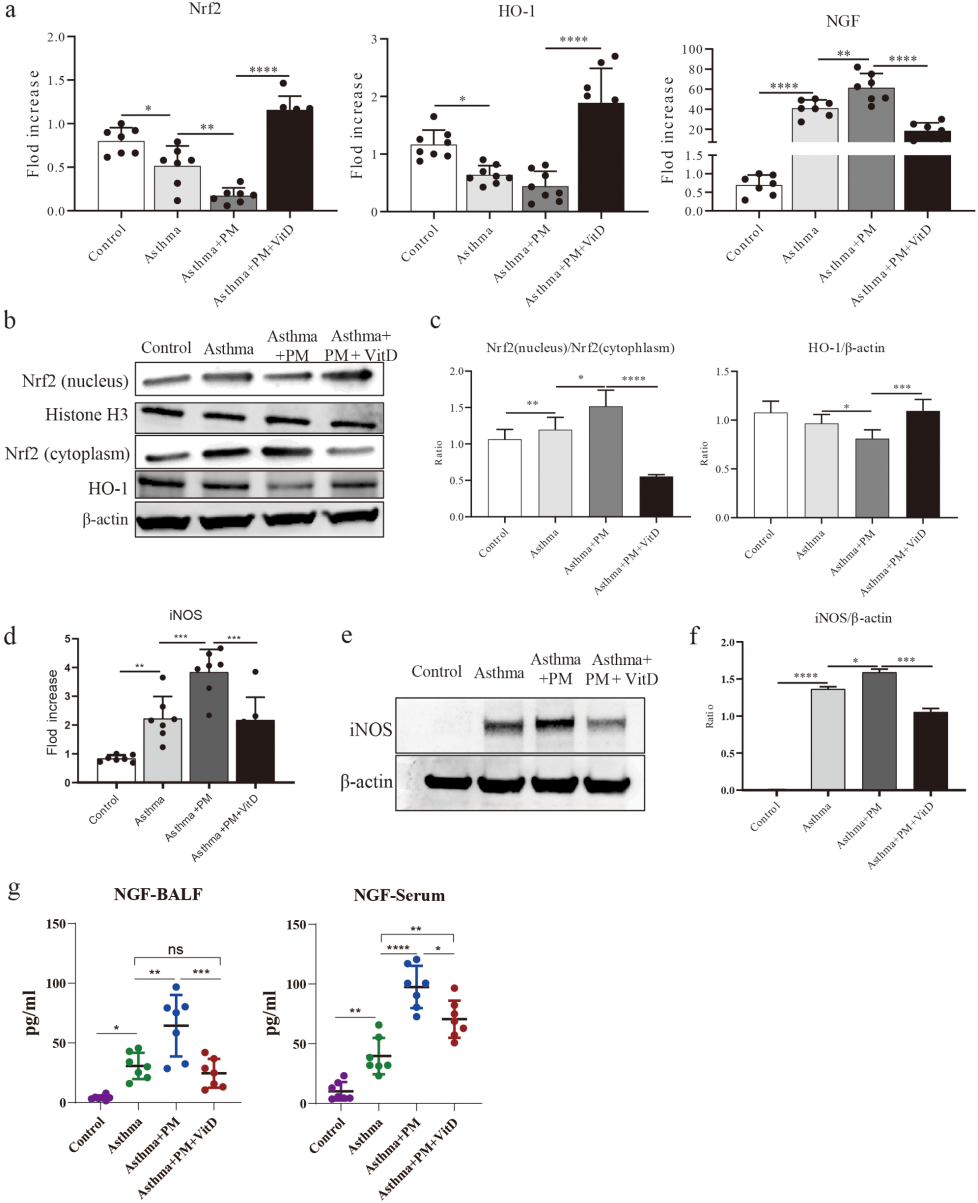
Vitamin D activates the Nrf2/HO-1 signaling pathway in mouse asthma. (**a**) qPCR was used to analyze Nrf2, HO-1 and NGF’s gene expression levels in lung tissue. (**b**) immunoblot analysis of the protein expression of Nrf2 and HO-1 in lung tissue. (**c**) gray-scale values count the relative protein expression of Nrf2 (nucleus) relative to Nrf2 (cytoplasm) and HO-1 relative to β-actin. (**d**) qPCR analysis of iNOS gene expression level in lung tissue. (**e**) immunoblot analysis of the protein expression of iNOS in lung tissue. (**f**) gray-scale values count the relative protein expression of iNOS relative to β-actin. (**g**) ELISA analysis of the NGF protein expression levels in BALF and serum. (* *p* < 0.05, ** *p* < 0.01, *** *p* < 0.001, and **** *p* < 0.0001)

Activation of the Nrf2/HO-1 signaling pathway can exert a protective effect on the inflammatory response in bronchial asthma [29]. So, the effects of vitamin D on Nrf2/HO-1 expression in the asthma mouse model were further evaluated. qPCR results showed that Nrf2 and HO-1 gene expression levels in the lung tissue of the Asthma group were significantly lower compared to the Control group. Additionally, in the Asthma + PM group, the gene expression levels were significantly lower than in the Asthma group. In contrast, the gene expression levels in the Asthma + PM + Vit D group were higher than in the Asthma group and significantly higher than in the Asthma + PM group. Furthermore, the expression of NGF gene was significantly up-regulated in both the Asthma and the Asthma + PM group at first. Of note, NGF expression was significantly decreased after vitamin D administration (Fig. 3a). WB and ELISA were used to measure the expression of Nrf2/HO-1 and NGF proteins in lung tissue, respectively. The results showed that Nrf2/HO-1’s expression decreased and NGF’s expression increased in the Asthma group, while vitamin D administration restored the gene expression levels (Fig. 3b, 3c, 3g). This indicates that vitamin D may activate the Nrf2/HO-1 signaling pathway in a PM-exposed asthma model, thereby reducing NGF’s expression.

HO-1 is a rate-limiting enzyme in heme degradation, that regulates redox reactions and has cytoprotective effects against oxidative stress-induced cellular damage. Since NO-producing iNOS is an important marker for oxidative stress pathway activation particularly induced by pollution exposure and allergic responses [30]. Therefore detecting the expression of iNOS in response to vitamin D or and PM can flank the activation of the HO-1 pathway. Since, iNOS is expressed in lung airway epithelial cells and macrophages [31], we performed qPCR and WB to detect iNOS expression in lung tissues. The results showed that iNOS shares the same expression trend with NGF and has this exact opposite expression trend with Nrf2 and HO-1 (Fig. 3d-3f). This suggests that oxidative stress occurs in lung tissues when HO-1 is lowly expressed, that PM exposure significantly increases this oxidative stress, and that the addition of vitamin D significantly mitigates the oxidative stress.

### Vitamin D activates the Nrf2/HO-1 signaling pathway in PM-exposed ASMCs

**Fig. 4:**
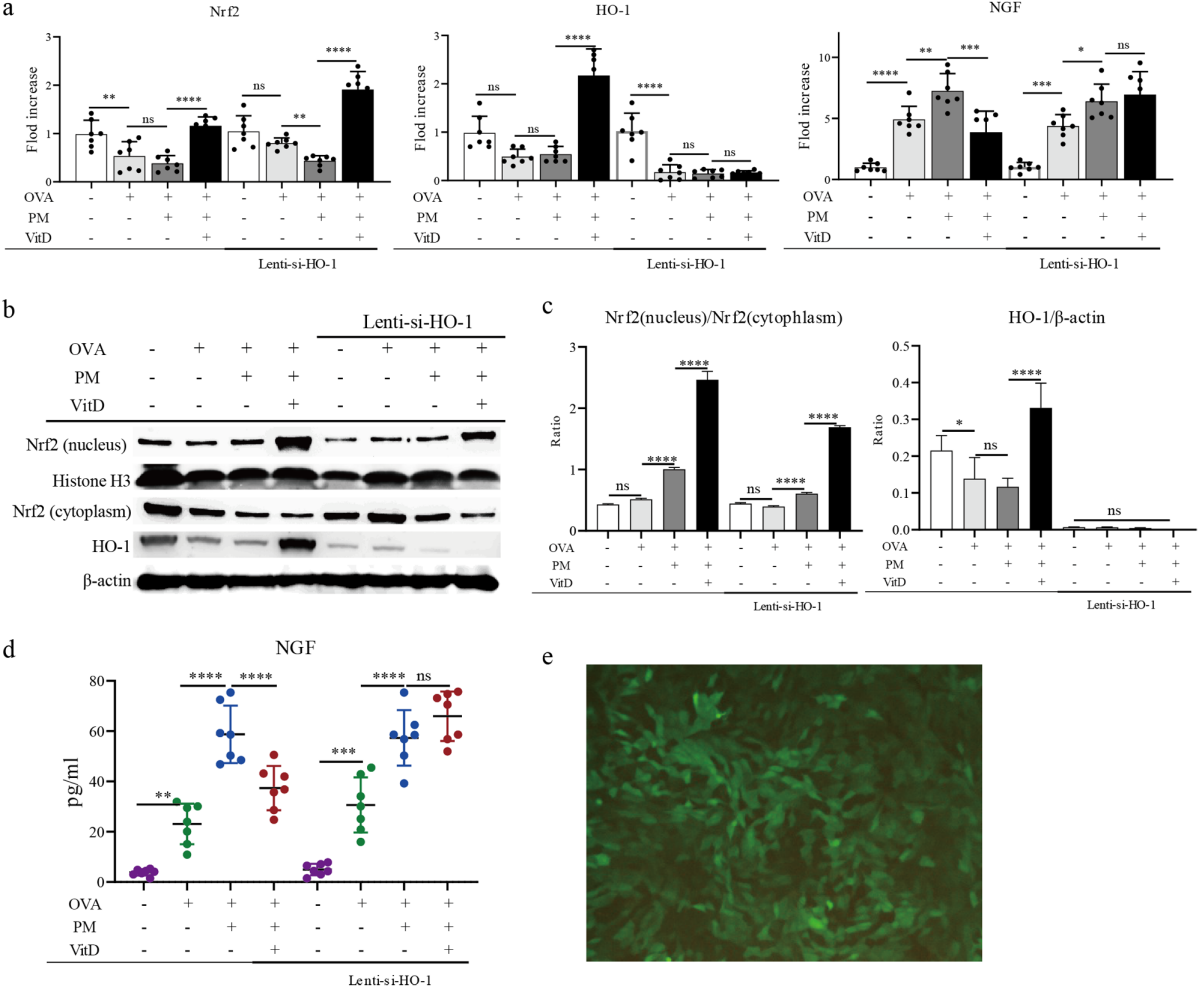
Vitamin D reversed Nrf2/HO-1’s low expression levels in ASMCs induced by PM or in ASMCs treated with HO-1 shRNA lentiviral vector. (**a**) qPCR was used to analyze Nrf2/HO-1 and NGF’s gene expression levels. (**b**) immunoblot analysis of the protein expression levels of Nrf2 and HO-1 on ASMCs treated using different substances. (**c**) gray-scale values count the relative protein expression of Nrf2 (nucleus) relative to Nrf2 (cytoplasm) and HO-1 relative to β-actin in ASMCs. (**d**) After treating ASMCs with different substances, ELISA was used to analyze NGF’s protein expression level in cell supernatants. (**e**) The Lenti-sh-HO-1 stable cell line. (* *p* < 0.05, ** *p* < 0.01, *** *p* < 0.001, and **** *p* < 0.0001)

I*n vivo* experiments preliminarily indicated that vitamin D might activate the Nrf2/HO-1 signaling pathway in the PM-exposed asthma model, thereby reducing NGF’s expression. To prove the protective effect of the vitamin D-mediated Nrf2/HO-1 pathway on asthma, we performed in vitro experiments. Specifically, 10^− 7^ mol/L vitamin D, 100 µg/ml OVA, and 100 µg/ml PM were co-incubated with ASMCs or Lenti-si-HO-1 ASMCs for 24 h, and then Nrf2/HO-1 expression levels were detected. Furthermore, we tested different vitamin D concentrations on ASMCs, and the optimal concentration and time of action were 10^− 7^mol/l and 24 h, respectively (shown in Supplementary Fig. 1). The results showed that OVA and PM, respectively, reduced Nrf2/HO-1 and increased NGF’s expression, while vitamin D reversed the process. In the HO-1 knockdown cell line of Lenti-si-HO-1 ASMCs, OVA and PM reduced Nrf2’s expression, while HO-1 and NGF’s expression were unchanged. These results suggest that vitamin Dreverses Nrf2’s expression, but does not affect HO-1 and NGF’s expression (Fig. 4).

## Discussion

This study shows that vitamin D effectively attenuates airway inflammation in PM-exposed asthmatic mice. Concretely, it ameliorates alveolar wall thickening, decreases mucus secretion without affecting the loss of alveolar structure, and reduces inflammatory cell infiltration. Of note, our in vivo, and in vitro molecular mechanism experiments have shown that vitamin D activates the Nrf2-HO-1 pathway and inhibits NGF’s expression. These results suggest that vitamin D supplementation in asthmatic mice activates the Nrf2-HO-1 pathway and suppresses NGF expression, thereby alleviating airway inflammation by reversing damage caused by PM exposure.

Recently, there has been increasing interest in the therapeutic use of vitamin D in asthma, and numerous reports have emerged on the adjuvant therapeutic effects of vitamin D supplementation in asthma. Based on multiple randomized, double-blind, parallel, placebo-controlled clinical trials, participants were randomly assigned to studies in which they took either a daily placebo capsule or a daily dose of vitamin D3 (e.g., 4,000 IU). The data from the Meta-analysis showed that vitamin D supplementation reduced the rate of acute asthma exacerbations requiring systemic corticosteroid therapy, while the duration of asthma exacerbations remained unchanged [18, 32]. Brustad et al. showed that supplementation with standard or high doses of vitamin D during pregnancy might have a protective effect against the onset of wheezing in children aged three years; however, this protective effect was not associated with the risk of developing asthma in six years old children [33]. In contrast, Lu et al. indicated that adequate vitamin D supplementation during pregnancy in mothers with asthma might provide good protection for their offspring by the age of three years [34]. Nevertheless, no consensus has been reached to date regarding the positive effects of vitamin D interventions on asthma. Specifically, other studies on diet and asthma showed that a higher vitamin D intake reduced the risk of wheezing in children [35, 36]. Remarkably, in their review, Garcia-Larsen et al. indicated that the protective effects of dietary antioxidants in asthma were more pronounced in childhood and were proportional to the duration of antioxidant use [37]. Discrepancies in the findings of the above observational studies are perhaps due to insufficient sample size and differences in patients’ baseline data.

Regardless of the controversies arising in epidemiological studies, more scholars favor the protective effect of vitamin D on asthma [32, 38]. Of note, numerous basic studies consistently show that vitamin D has anti-inflammatory and antioxidant protective effects against asthma. Specifically, it has been shown that vitamin D alters the development of immune cells, prolongs the survival and migration of eosinophils, and inhibits fibroblast secretion of fibrogenic mediators [39]. Vitamin D has also been proven to stabilize alveolar type II cell DNA synthesis [40, 41] and to modify alveolarization [42]. Other studies have shown that the active metabolite of vitamin D is protective in asthmatic rodents [43, 44]. Based on these findings, more conclusive evidence is needed to understand the exact mechanisms of vitamin D’s anti-inflammatory and antioxidant activities. Therefore, hopefully, more in-depth basic studies will be conducted.

Epidemiological studies have shown that children with vitamin D deficiency are susceptible to exposure to traffic-related particulate pollutants. Bolcas et al. [12] reported that maintaining the normal level of vitamin D during the early life of an asthma mouse model has several effects. Specifically, it can reduce the number of Th1/Th17 cells in the lung, inhibit airway hyperresponsiveness, and significantly reduce the aggravation of allergic asthma related to diesel exhaust particulate (DEP) exposure. The authors showed that vitamin D might effectively prevent asthma development in people exposed to traffic-related particulate pollutants. However, two additional studies independently reported that vitamin D supplementation did not significantly improve the duration of severe asthma attacks [18, 32] but significantly reduced the occurrence and exacerbation of asthma [18]. Of note, in the present study, asthma was induced by OVA challenge and aggravated by PM exposure, causing an increased count of eosinophils, airway inflammatory cell infiltration, and the upregulation of Th2/Th1 ratio in the lung. Importantly, vitamin D treatment significantly reversed these changes. Interestingly, Rigo et al. [45] reported that vitamin D3 is involved in regulating the innate immune defenses of the airway epithelium, and its deficiency contributes to the exacerbation of OVA-induced airway disease in asthmatic mice [46]. Furthermore, another study showed that 1,25-dihydroxyvitamin D3 reduced airway inflammation in asthmatic mice [47]. These results confirm that vitamin D3 protects the airways from OVA-induced inflammatory damage.

Vitamin D protects against airway disease through antioxidant and immunomodulatory pathways [48, 49]. Vitamin D is protective in asthmatics against environmental particulate matter exposure. A cohort study of personal vitamin D status in a predominantly black urban of children shows children with asthma who are vitamin D deficient are more likely to develop sensitivity to PM_2.5_ [50]. This study reveals that individual vitamin D status in asthmatic children was associated with respiratory symptoms caused by indoor PM_2.5_ exposure. Serum 25-OH vitamin D levels below 15.5 ng/mL in asthmatic children increased individual susceptibility to the respiratory effects of indoor PM_2.5_, especially in obese children. In contrast, higher serum 25-OH vitamin D levels were protective against symptoms associated with indoor PM_2.5_ exposure. Under exposure to organic dust, pro-inflammatory responses were attenuated in mice fed a high-dose vitamin D diet and in cells pretreated with vitamin D compared to mice fed a low-dose vitamin D diet [51]. These results suggest that vitamin D supplementation protects against asthma under PM exposure and that this protection is dose-dependent.

Remarkably, in our study, there was a downregulation of HO-1 in the OVA-induced allergic asthma mouse model. As a protective protein, several studies have reported increased HO-1 expression in the airways of asthmatics [52, 53]. Interestingly, in a mouse model of asthma, HO-1 expression increased in the airway lumen and bronchial submucosa of antigen-challenged mice [54, 55]. On the contrary, consistent with our findings, additional studies showed that HO-1’s expression did not significantly increase in asthmatic mice lung tissues [56, 57] and tended to be under-expressed in OVA-stimulated airway epithelial cells [56]. During antigen presentation, dendritic cells (DC) cells with high HO-1 expression secrete high levels of IL-10 and TGF-β, and low levels of IL-12 and IL-23. In turn, the differentiation of naive T cells to Treg cells is promoted, and the differentiation of naive T cells to Th2 and Th17 cells is inhibited [58, 59], preventing further inflammation. Such observations suggest that high HO-1 expression and anti-inflammatory effects may occur during the initial immune phase. This finding is consistent with our previous study showing that the Th1/Th2 ratio remains relatively constant in OVA-induced mice at the same time, Treg/Th17 changes rapidly, eventually leading to pathological lesions in the respiratory tract [60]. However, to confirm and validate this hypothesis, more relevant experiments need to be conducted.

Our study bridges the gap between observational epidemiological data and experimental animal models by investigating the intricate signaling pathways through which vitamin D exerts its protective effects in PM-exposed asthma. Through our comprehensive approach, we provide valuable insights into the biological significance of vitamin D and its potential role in mitigating the deleterious impact of particulate matter on asthma. While, Nrf2 maintains cellular redox homeostasis by regulating the downstream antioxidant HO-1 [20, 21], thereby providing cytoprotection against oxidative stress-induced cellular damage. In this article, from an animal model, we detected a significant increase in the expression of iNOS in asthmatic lung tissues in response to PM, which was significantly down-regulated after administration of Vit D treatment. Hence we suggest that this pathway also touches a significant oxidative stress response. The process of inflammation was explored in our paper only from the pathway of NGF, however, not much validation was made about the factors related to oxidative stress. For example, the expression of iNOS and ROS and the release of NO under Vit D or PM treatment could be examined in a macrophage cell system or using an airway epithelial cell system. In addition, the reason that the metrics in the Asthma group and the Asthma + PM + Vit D group were not compared in this paper is that the variability of the metrics between the two groups was inconsistent in our results, e.g., the difference in NGF was significant in serum but not in BALF. Therefore, our results do not definitively conclude whether vitamin D can completely alleviate the inflammatory response in asthma due to PM. And to clarify this mechanism, another study is needed, such as the exploration of the concentration gradient of administration and the exploration of the time gradient of administration. Consequently, this research direction is a long way to go, and a lot of basic research is still needed to clarify the inflammatory mechanism of this pathway, and we will conduct experiments in this direction in the future in order to clarify the more specific mechanism of action.

## Conclusion

Our study shows that vitamin D is involved, in vivo, in regulating inflammation in PM-exposed asthma. Additionally, we demonstrate that vitamin D plays a protective role, in vitro, in PM-exposed allergic asthma by regulating the Nrf2/HO-1 and NGF signaling pathways. Our study provides mechanistic evidence to support that vitamin D supplementation can mitigate the deleterious effects of particulate matter on asthma.

### Electronic supplementary material

Below is the link to the electronic supplementary material.

**Supplementary Material 1**: Optimization of vitamin D’s optimal concentration and acting time

**Supplementary Material 2**: Different MOI gradients and infection enhancement solutions for cell infection efficiency

**Supplementary Material 3**: Inhibited efficiency of HO-1 sh-RNAs

**Supplementary Material 4**: Supplementary Methods, Table and Multiple exposure images

## Data Availability

The datasets generated and analysed during the current study are available from the correspondence author on reasonable request.

## Abbreviations

PM: Particulate Matter
ASMCs: Mouse airway smooth muscle cells
BALF: Bronchoalveolar lavage fluid
OVA: Ovalbumin
NGF: Nerve growth factor
HO-1: Heme oxygenase-1
NQO1: NAD(P)H dehydrogenase quinone 1
SOD: Superoxide dismutase
ROS: Reactive oxygen species
XMULAC: Xiamen University Laboratory Animal Center
i.p.: Intraperitoneal
Lenti-si-HO-1: Lentivirus expressing a HO-1 shRNA
ANOVA: One-way analysis of variance
DC: Dendritic cells

## References

[1] Cloutier MM, Dixon AE, Krishnan JA, Lemanske RF, Pace W, Schatz M (2020). Managing asthma in adolescents and adults: 2020 Asthma Guideline Update from the National Asthma Education and Prevention Program. JAMA.

[2] Dana L, Yann G, Béatrice LS, Fatiha EG, Véronique B, Lamia BT, Neela G, Robert B, Heidi M, Kurt S (2013). The carcinogenicity of outdoor air pollution. Lancet Oncol.

[3] Edginton S, O’Sullivan DE, King WD, Research MLJE. The effect of acute outdoor air pollution on peak expiratory flow in individuals with asthma: a systematic review and meta-analysis. 2021, 192:110296. 10.1016/j.envres.2020.110296 33031812

[4] Faraji M, Mohammadi A, Ajmi N, Fallahnezhad M, Sabetkish M, Kazemnejad N, Shoormasti A, Fazlollahi RS, Pourpak MR, Moin Z. M: Exposure to ambient air pollution and prevalence of asthma in adults. 2021.

[5] Hsu HH, Chiu YH, Coull BA, Kloog I, Schwartz J, Lee A, Wright RO, Wright RJ (2015). Prenatal Particulate Air Pollution and Asthma Onset in Urban Children. Identifying sensitive Windows and Sex differences. Am J Respiratory Crit Care Med.

[6] Movassagh H, Prunicki M, Smith E, Dunham D, Nadeau KJJA, Immunology C. Dysregulation of circulating Monocytes is Associated with exposure to Air Pollution and Asthma in Children. 2021, 147(2):AB175.

[7] El-Sharkawy A, Malki A. Vitamin D signaling in inflammation and Cancer: Molecular mechanisms and therapeutic implications. Molecules 2020, 25(14). 10.3390/molecules25143219 PMC7397283 32679655

[8] Bikle D, Christakos S (2020). New aspects of vitamin D metabolism and action - addressing the skin as source and target. Nat Reviews Endocrinol.

[9] Campolina-Silva G, Andrade A, Couto M, Bittencourt-Silva PG, Queiroz-Junior CM, Lacerda LSB, Chaves IM, de Oliveira LC, Marim FM, Oliveira CA et al. Dietary vitamin D mitigates Coronavirus-Induced Lung inflammation and damage in mice. Viruses 2023, 15(12). 10.3390/v15122434 PMC10748145 38140675

[10] Trombetta AC, Smith V, Gotelli E, Ghio M, Paolino S, Pizzorni C, Vanhaecke A, Ruaro B, Sulli A, Cutolo M (2017). Vitamin D deficiency and clinical correlations in systemic sclerosis patients: a retrospective analysis for possible future developments. PLoS ONE.

[11] Mitchell DM, Henao MP, Finkelstein JS, Burnett-Bowie SA (2012). Prevalence and predictors of vitamin D deficiency in healthy adults. Endocr Practice: Official J Am Coll Endocrinol Am Association Clin Endocrinologists.

[12] Bolcas PE, Brandt EB, Zhang Z, Biagini Myers JM, Ruff BP, Khurana Hershey GK (2019). Vitamin D supplementation attenuates asthma development following traffic-related particulate matter exposure. J Allergy Clin Immunol.

[13] Kaur N, Kumar V, Singh J, Jain H, Paras P, Kaur N, Sareen AK (2023). Assessment of the relation between asthma severity and serum vitamin D levels: a cross-sectional study. Cureus.

[14] Mukherjee S, Lindell DM, Berlin AA, Morris SB, Shanley TP, Hershenson MB, Lukacs NW (2011). IL-17-induced pulmonary pathogenesis during respiratory viral infection and exacerbation of allergic disease. Am J Pathol.

[15] Greiller CL, Martineau AR (2015). Modulation of the immune response to respiratory viruses by vitamin D. Nutrients.

[16] Nanzer AM, Chambers ES, Ryanna K, Richards DF, Black C, Timms PM, Martineau AR, Griffiths CJ, Corrigan CJ, Hawrylowicz CM (2013). Enhanced production of IL-17A in patients with severe asthma is inhibited by 1α,25-dihydroxyvitamin D3 in a glucocorticoid-independent fashion. J Allergy Clin Immunol.

[17] Brehm JM, Acosta-Pérez E, Klei L, Roeder K, Barmada M, Boutaoui N, Forno E, Kelly R, Paul K, Sylvia J (2012). Vitamin D insufficiency and severe asthma exacerbations in Puerto Rican children. Am J Respir Crit Care Med.

[18] Jolliffe DA, Greenberg L, Hooper RL, Griffiths CJ, Camargo CA, Kerley CP, Jensen ME, Mauger D, Stelmach I, Urashima M (2017). Vitamin D supplementation to prevent asthma exacerbations: a systematic review and meta-analysis of individual participant data. The Lancet Respiratory Medicine.

[19] Rosser F, Brehm JM, Forno E, Acosta-Pérez E, Kurland K, Canino G, Celedón JC (2014). Proximity to a major road, vitamin D insufficiency, and severe asthma exacerbations in Puerto Rican children. Am J Respir Crit Care Med.

[20] Hafez HM, Ibrahim MA, Zedan MZ, Hassan M, Hassanein H (2019). Nephroprotective effect of cilostazol and verapamil against thioacetamide-induced toxicity in rats may involve Nrf2/HO-1/NQO-1 signaling pathway. Toxicol Mech Methods.

[21] Fredenburgh LE, Perrella MA, Mitsialis SA (2007). The role of heme oxygenase-1 in pulmonary disease. Am J Respir Cell Mol Biol.

[22] Kuribayashi K, Iida S, Nakajima Y, Funaguchi N, Tabata C, Fukuoka K, Fujimori Y, Ihaku D, Nakano T (2015). Suppression of heme oxygenase-1 activity reduces airway hyperresponsiveness and inflammation in a mouse model of asthma. J Asthma: Official J Association Care Asthma.

[23] Mishina K, Shinkai M, Shimokawaji T, Nagashima A, Hashimoto Y, Inoue Y, Inayama Y, Rubin BK, Ishigatsubo Y, Kaneko T (2015). HO-1 inhibits IL-13-induced goblet cell hyperplasia associated with CLCA1 suppression in normal human bronchial epithelial cells. Int Immunopharmacol.

[24] Xia M, Viera-Hutchins L, Garcia-Lloret M, Noval Rivas M, Wise P, McGhee SA, Chatila ZK, Daher N, Sioutas C, Chatila TA (2015). Vehicular exhaust particles promote allergic airway inflammation through an aryl hydrocarbon receptor-notch signaling cascade. J Allergy Clin Immunol.

[25] Sun L, Fu J, Lin SH, Sun JL, Xia L, Lin CH, Liu L, Zhang C, Yang L, Xue P (2020). Particulate matter of 2.5 µm or less in diameter disturbs the balance of T(H)17/regulatory T cells by targeting glutamate oxaloacetate transaminase 1 and hypoxia-inducible factor 1α in an asthma model. J Allergy Clin Immunol.

[26] Zhang H, Wang, Zhihui S. Xiaohan, Ren, reports LJMm: the protective role of vitamin D3 in a murine model of asthma via the suppression of TGF-/Smad signaling and activation of the Nrf2/HO-1 pathway. 2016. 10.3892/mmr.2016.5563 PMC4991747 27484042

[27] Barrios J, Ai X (2018). Neurotrophins in Asthma. Curr Allergy Asthma Rep.

[28] Dagnell C, Kemi C, Klominek J, Eriksson P, Sköld CM, Eklund A, Grunewald J, Olgart Höglund C (2007). Effects of neurotrophins on human bronchial smooth muscle cell migration and matrix metalloproteinase-9 secretion. Translational Research: The Journal of Laboratory and Clinical Medicine.

[29] Liu X, Yu D, Wang T (2016). Sappanone A attenuates allergic Airway inflammation in Ovalbumin-Induced Asthma. Int Arch Allergy Immunol.

[30] Zhu XM, Wang Q, Xing WW, Long MH, Fu WL, Xia WR, Jin C, Guo N, Xu DQ, Xu DG (2018). PM2.5 induces autophagy-mediated cell death via NOS2 signaling in human bronchial epithelium cells. Int J Biol Sci.

[31] Switzer CH, Cho HJ, Eykyn TR, Lavender P, Eaton P (2022). NOS2 and S-nitrosothiol signaling induces DNA hypomethylation and LINE-1 retrotransposon expression. Proc Natl Acad Sci U S A.

[32] Forno E, Bacharier LB, Phipatanakul W, Guilbert TW, Cabana MD, Ross K, Covar R, Gern JE, Rosser FJ, Blatter J (2020). Effect of vitamin D3 supplementation on severe asthma exacerbations in children with asthma and Low Vitamin D levels: the VDKA Randomized Clinical Trial. JAMA.

[33] Brustad N, Eliasen AU, Stokholm J, Bønnelykke K, Bisgaard H, Chawes BL (2019). High-dose vitamin D supplementation during pregnancy and asthma in offspring at the age of 6 years. JAMA.

[34] Lu M, Litonjua AA, O’Connor GT, Zeiger RS, Bacharier L, Schatz M, Carey VJ, Weiss ST, Mirzakhani H (2021). Effect of early and late prenatal vitamin D and maternal asthma status on offspring asthma or recurrent wheeze. J Allergy Clin Immunol.

[35] Nurmatov U, Devereux G, Sheikh A (2011). Nutrients and foods for the primary prevention of asthma and allergy: systematic review and meta-analysis. J Allergy Clin Immunol.

[36] Garcia-Marcos L, Castro-Rodriguez JA, Weinmayr G, Panagiotakos DB, Priftis KN, Nagel G (2013). Influence of Mediterranean diet on asthma in children: a systematic review and meta-analysis. Pediatr Allergy Immunology: Official Publication Eur Soc Pediatr Allergy Immunol.

[37] Garcia-Larsen V, Del Giacco SR, Moreira A, Bonini M, Charles D, Reeves T, Carlsen KH, Haahtela T, Bonini S, Fonseca J (2016). Asthma and dietary intake: an overview of systematic reviews. Allergy.

[38] Pfeffer PE, Hawrylowicz CM (2018). Vitamin D in Asthma: mechanisms of action and considerations for clinical trials. Chest.

[39] Poon AH, Mahboub B, Hamid Q (2013). Vitamin D deficiency and severe asthma. Pharmacol Ther.

[40] Edelson JD, Chan S, Jassal D, Post M, Tanswell AK (1994). Vitamin D stimulates DNA synthesis in alveolar type-II cells. Biochim Biophys Acta.

[41] Phokela SS, Peleg S, Moya FR, Alcorn JL (2005). Regulation of human pulmonary surfactant protein gene expression by 1alpha,25-dihydroxyvitamin D3. Am J Physiol Lung Cell Mol Physiol.

[42] Nadeau K, Montermini L, Mandeville I, Xu M, Weiss ST, Sweezey NB, Kaplan F (2010). Modulation of Lgl1 by steroid, retinoic acid, and vitamin D models complex transcriptional regulation during alveolarization. Pediatr Res.

[43] Lai G, Wu C, Hong J, Song Y (2013). 1,25-Dihydroxyvitamin D(3) (1,25-(OH)(2)D(3)) attenuates airway remodeling in a murine model of chronic asthma. J Asthma: Official J Association Care Asthma.

[44] Zhou Y, Zhou X, Wang X (2008). 1,25-Dihydroxyvitamin D3 prevented allergic asthma in a rat model by suppressing the expression of inducible nitric oxide synthase. Allergy and Asthma Proceedings.

[45] Rigo I, McMahon L, Dhawan P, Christakos S, Yim S, Ryan LK, Diamond G (2012). Induction of triggering receptor expressed on myeloid cells (TREM-1) in airway epithelial cells by 1,25(OH)_2_; vitamin D_3_. Innate Immun.

[46] Zhou Y, Xue Y, Bao A, Han L, Bao W, Xia C, Tian X, Zhang M (2021). Effect of vitamin D Deficiency and Supplementation in Lactation and Early Life on Allergic Airway Inflammation and the expression of Autophagy-related genes in an Ovalbumin Mouse Model. J Inflamm Res.

[47] Qiu YY, Zhou XY, Qian XF, Wu YX, Qin C, Bian T (2017). 1,25-dihydroxyvitamin D3 reduces mouse airway inflammation of neutrophilic asthma by transcriptional modulation of interleukin-17A. Am J Translational Res.

[48] Sypniewska G, Krintus M, Fulgheri G, Siodmiak J, Kuligowska-Prusinska M, Stepien-Jaszowska B, Staszak-Kowalska R, Zawadzka-Krajewska A, Kierat S, Bergmann K (2017). 25-Hydroxyvitamin D, biomarkers of eosinophilic inflammation, and airway remodeling in children with newly diagnosed untreated asthma. Allergy and Asthma Proceedings.

[49] Szentpetery SE, Han YY, Brehm JM, Acosta-Pérez E, Forno E, Boutaoui N, Canino G, Alcorn JF, Celedón JC (2018). Vitamin D insufficiency, plasma cytokines, and severe asthma exacerbations in school-aged children. J Allergy Clin Immunol Pract.

[50] Bose S, Diette GB, Woo H, Koehler K, Romero K, Rule AM, Detrick B, Brigham E, McCormack MC, Hansel NN (2019). Vitamin D status modifies the response to indoor particulate matter in obese Urban children with asthma. J Allergy Clin Immunol Pract.

[51] Golden GA, Wyatt TA, Romberger DJ, Reiff D, McCaskill M, Bauer C, Gleason AM, Poole JA (2013). Vitamin D treatment modulates organic dust-induced cellular and airway inflammatory consequences. J Biochem Mol Toxicol.

[52] Kim SM, Ryu HW, Kwon OK, Hwang D, Kim MG, Min JH, Zhang Z, Kim SY, Paik JH, Oh SR (2021). Callicarpa japonica Thunb. Ameliorates allergic airway inflammation by suppressing NF-κB activation and upregulating HO-1 expression. J Ethnopharmacol.

[53] Harju T, Soini Y, Pääkkö R, Kinnula VL (2002). Up-regulation of heme oxygenase-I in alveolar macrophages of newly diagnosed asthmatics. Respir Med.

[54] Kitada O, Kodama T, Kuribayashi K, Ihaku D, Fujita M, Matsuyama T, Sugita M (2001). Heme oxygenase-1 (HO-1) protein induction in a mouse model of asthma. Clin Experimental Allergy: J Br Soc Allergy Clin Immunol.

[55] Almolki A, Taillé C, Martin GF, Jose PJ, Zedda C, Conti M, Megret J, Henin D, Aubier M, Boczkowski J (2004). Heme oxygenase attenuates allergen-induced airway inflammation and hyperreactivity in guinea pigs. Am J Physiol Lung Cell Mol Physiol.

[56] Lv J, Su W, Yu Q, Zhang M, Di C, Lin X, Wu M, Xia Z (2018). Heme oxygenase-1 protects airway epithelium against apoptosis by targeting the proinflammatory NLRP3-RXR axis in asthma. J Biol Chem.

[57] Lin XL, Lv JJ, Lv J, Di CX, Zhang YJ, Zhou T, Liu JL, Xia ZW (2017). Heme oxygenase-1 directly binds STAT3 to control the generation of pathogenic Th17 cells during neutrophilic airway inflammation. Allergy.

[58] Wong TH, Chen HA, Gau RJ, Yen JH, Suen JL (2016). Heme Oxygenase-1-Expressing dendritic cells promote Foxp3 + Regulatory T Cell differentiation and induce less severe airway inflammation in murine models. PLoS ONE.

[59] Pae HO, Oh GS, Choi BM, Chae SC, Chung HT (2003). Differential expressions of heme oxygenase-1 gene in CD25- and CD25 + subsets of human CD4 + T cells. Biochem Biophys Res Commun.

[60] Wu J, Ge D, Zhong T, Chen Z, Zhou Y, Hou L, Lin X, Hong J, Liu K, Qi H (2020). IRF4 and STAT3 activities are associated with the imbalanced differentiation of T-cells in responses to inhalable particulate matters. Respir Res.

