# Supplementary figures and images for: Unveiling the potent effect of vitamin D: harnessing Nrf2/HO-1 signaling pathways as molecular targets to alleviate urban particulate matter-induced asthma inflammation
Source: BMC Pulm Med. 2024 Jan 25;24:55. doi: 10.1186/s12890-024-02869-2 (PMC10809564; doi:10.1186/s12890-024-02869-2)

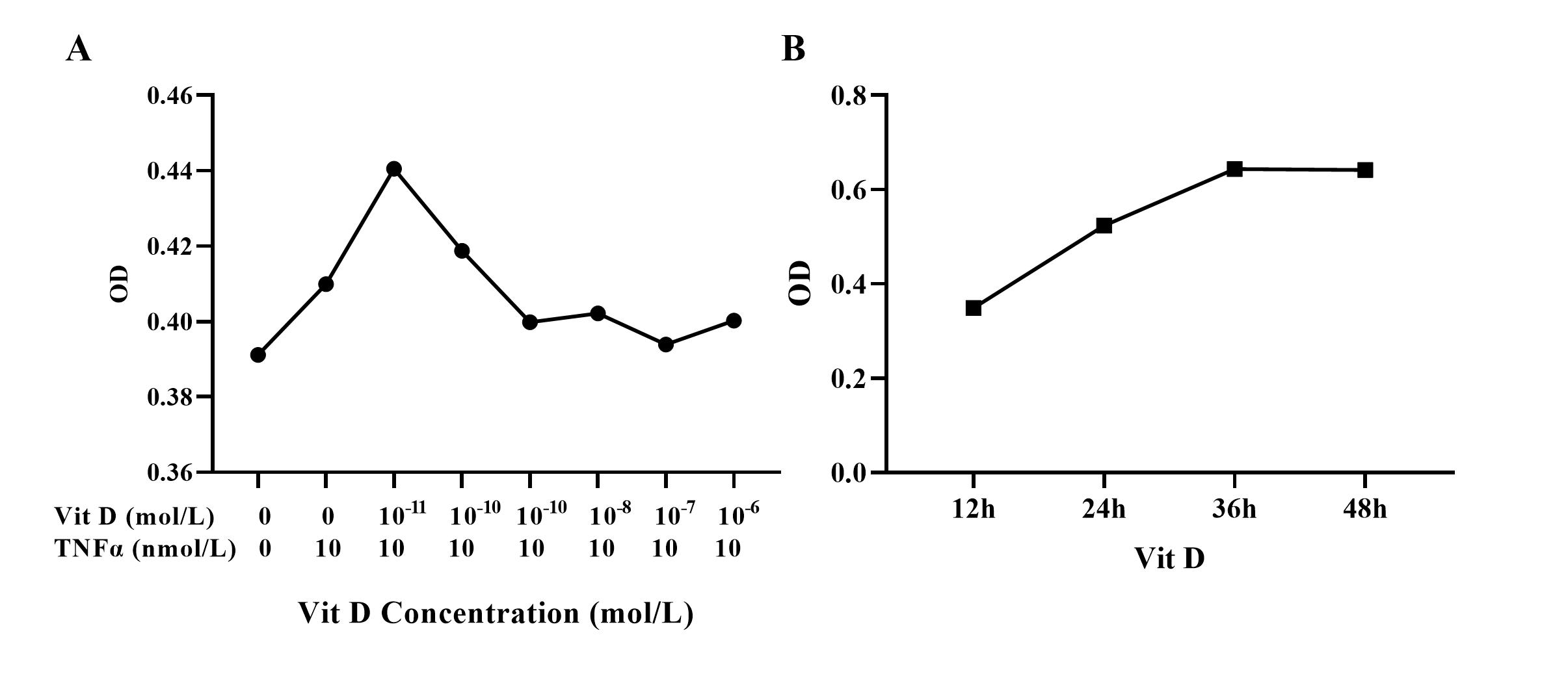

Supplement: Supplementary file 1 — Supplementary Material 1: Optimization of vitamin D’s optimal concentration and acting time [file 12890_2024_2869_MOESM1_ESM.jpg]

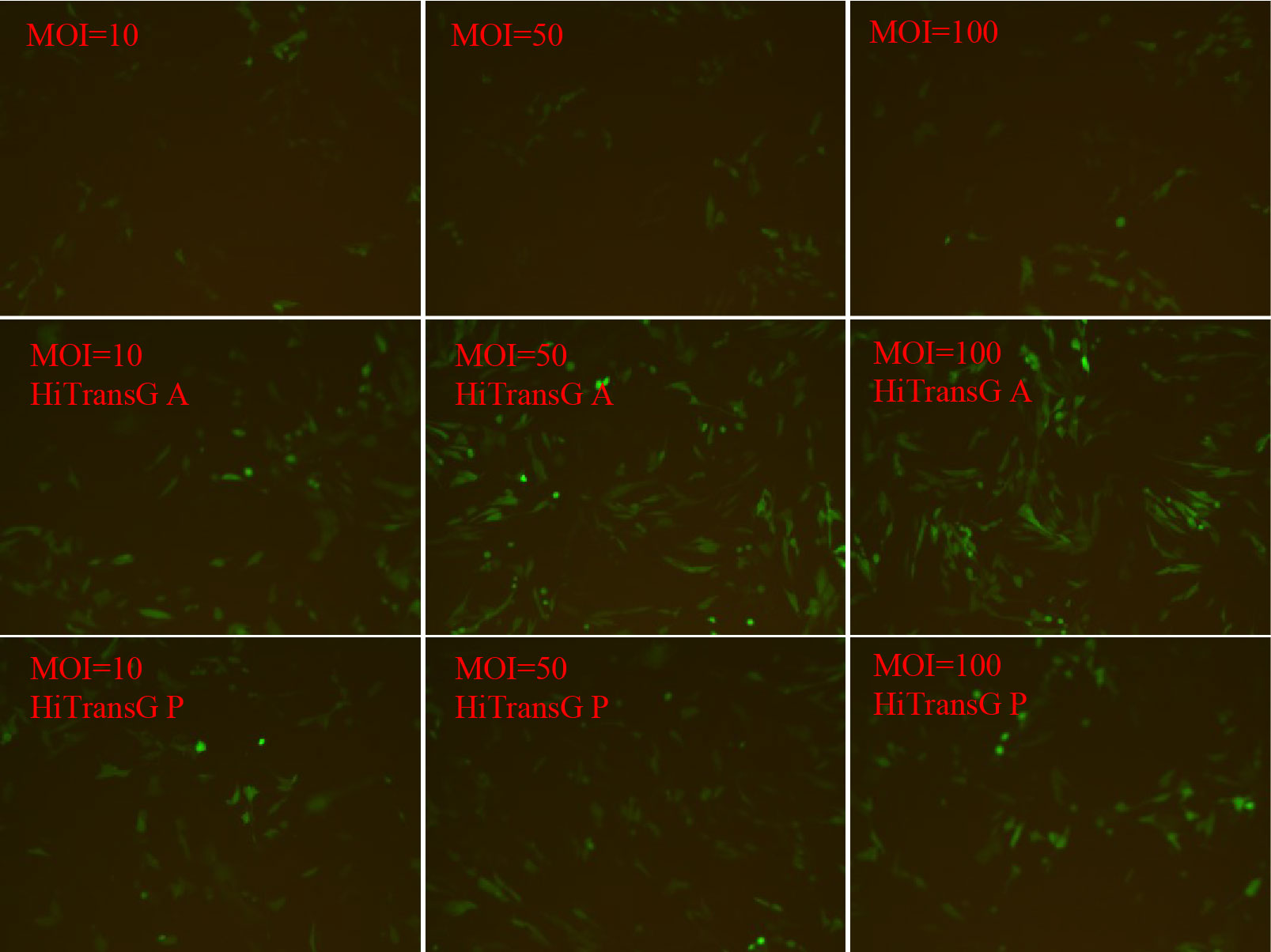

Supplement: Supplementary file 2 — Supplementary Material 2: Different MOI gradients and infection enhancement solutions for cell infection efficiency [file 12890_2024_2869_MOESM2_ESM.jpg]

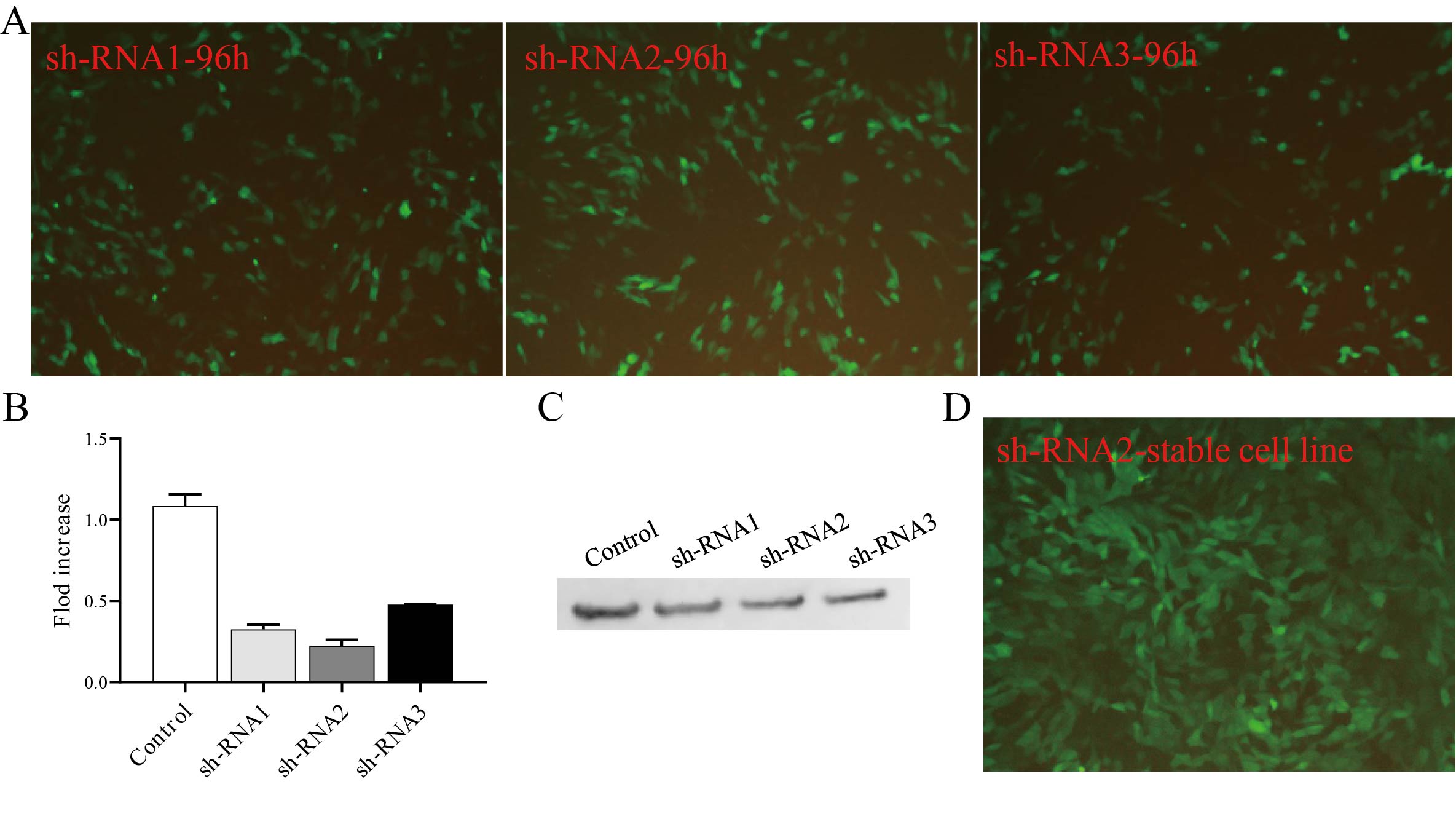

Supplement: Supplementary file 3 — Supplementary Material 3: Inhibited efficiency of HO-1 sh-RNAs [file 12890_2024_2869_MOESM3_ESM.jpg]
