# Supplementary material for: Unveiling the potent effect of vitamin D: harnessing Nrf2/HO-1 signaling pathways as molecular targets to alleviate urban particulate matter-induced asthma inflammation
Source: BMC Pulm Med. 2024 Jan 25;24:55. doi: 10.1186/s12890-024-02869-2 (PMC10809564; doi:10.1186/s12890-024-02869-2)
Supplement: Supplementary file 4 — Supplementary Material 4: Supplementary Methods, Table and Multiple exposure images [file 12890_2024_2869_MOESM4_ESM.docx]

Supplementary Material

**Unveiling the Potent Role of Vitamin D: Harnessing Nrf2/HO-1 Signaling Pathways as Molecular Targets to Alleviate Urban Particulate Matter-induced Asthma Inflammation**

Dandan Ge^a^ Qihong Chen^a^ Xiaohua Xie ^a^ Qiyuan Li^b^ Yungang Yang ^a^

*** Correspondence:** Yungang Yang: xmyyg@sina.com

# Methods

###### Blood, BALF, and lung tissue

Twenty-four hours after the last PM exposure, 0.5-0.8ml of retro-orbital blood was collected and clotted at room temperature for 2-4 hours. Then serum was isolated and stored at minus 80°C. After collecting retro-orbital blood, a tracheotomy was performed, and a cannula was inserted into the trachea. Ice-cold Saline (1ml) was instilled into the lungs, and BAL fluid was collected. The fluid was then centrifuged, and aliquots of supernatant were stored at minus 80°C. Lung tissue was fixed with formaldehyde, then immobilized, and paraffinized.

###### Lung tissue histopathology staining

Paraffin-embedded lung sections (4 μm) were stained with hematoxylin and eosin (H&E) or with periodic acid–schiff (PAS). Cellular infiltration around the blood vessels and distal fine bronchi was scored to evaluate the lung inflammation according to the following criteria: 0, no infiltrates; 1, few inflammatory cells; 2, a ring of inflammatory cells one cell layer deep; 3, a ring of inflammatory cells 2 to 4 cells deep; and 4, a ring of inflammatory cells greater than 4 cells deep [1]. A composite score was determined by adding the inflammatory scores for both vessels and airways. To further assess the inflammation in the lung, we evaluated the number and distribution of goblet cells through periodic acid–Schiff (PAS) staining of mucin granules.

Individual airways (bronchi/bronchioles) were scored for goblet cell hyperplasia according to the following scale: 0, no PAS-positive cells; 1, less than 5% PAS-positive cells; 2, 5% to 10% PAS-positive cells; 3, 10% to 25% PAS-positive cells; and 4, greater than 25% PAS-positive cells [2]. To reduce human random error, we are anonymizing the images and having three people in the lab do three counts each and taking the average of the three for statistical analysis.

###### ELISA

For in vitro cytokine concentration determination, supernatants from differentiated T cells were collected on day 3 of culture and analyzed by capture ELISA. For in vivo cytokine concentration determination, retro-orbital blood and bronchoalveolar lavage fluid (BALF) were collected from the model mice. BALF supernatants and serum were collected post-centrifugation and analyzed by capture ELISA. IL-1β, TNF-α, IL-6, and NGF ELISAs were performed using mouse kits per Novus Biologicals’s recommendations.

###### Quantitative real-time PCR

Total RNA was isolated from lung tissue using RNeasy Isolation Kit (Promega) and treated with DNAse (Qiagen) following the manufacturer’s instruction. Subsequently, cDNA synthesis was performed from 2µg of total RNA with FastQuant RT Kit (with gDNase, TIANGEN). Quantitative real-time PCR was performed using SYBR green–based reagents on the ViiA 7 Real-Time PCR System (Life Technologies). To this end, we used primer pairs targeting the cDNAs of Nrf2, Ho-1, NGF, and β-actin transcripts. iQT^M^SYBR Green Super mix (2x, Tiangen) and qPCR primers were purchased from Sangon Biotech (Shanghai Co., Ltd). The comparative threshold cycle (Ct) method normalized to β-actin was used to analyze relative changes in gene expression. The fold-change was calculated as 2^-ΔΔCt^ for negative ΔΔCt values (indicating a fold change increase) and -2^ΔΔCt^ for positive ΔΔCt values (indicating a fold change decrease). All qPCR reactions were run in duplicates, and the CT values were normalized to actin as a fold-induction over controls.

###### Western blotting

After grinding with a tissue grinder, lung tissue was lysed in RIPA buffer (phosphate-buffered saline containing 1% Triton X-100, 0.5% deoxycholate, 0.1% sodium dodecyl sulfate [SDS], 1 mM Na3VO4), complete protease inhibitors and protein phosphatase inhibitor complex (Roche). After clearing lysates by centrifugation (14000×g, 5min at 4°C), protein concentrations were determined by BCA assays. Twenty to 80μg of proteins were separated on an SDS–10% polyacrylamide electrophoresis (SDS-PAGE) gel and transferred to polyvinylidene difluoride (PVDF) membranes by electroblotting. Immunoreactive proteins were visualized by the ECL method (Pierce) and imaged with a Fujifilm LAS-4000 imager.

###### The electro-transferred membranes that were clipped prior to hybridisation with antibodies according to the marker's instructions, so some WB results will present different lengths. During gel imaging, different membranes with the same primary antibody will be gel imaged at the same time, therefore, several membranes may appear in the same picture at the same time.Optimization of vitamin D’s optimal concentration

ASMCs were taken in a logarithmic growth period, 6 × 10^3^ cells/wells were inoculated and incubated in a 96-well culture plate. After the cells fully adhered to the wall and stretched, they were synchronized. TNFα was then given. Cells were stimulated and divided into six subgroups. Specifically, 10^-6^ ~ 10^-11^ mol/L vitamin D were added to the cells, followed by a 24 hours incubation. Subsequently, CCK8 solution was added to each well, and cells were incubated for 1 hour. A 450 nm wavelength was then selected and the absorbance value of each well was measured with an automatic enzyme-linked detector. Finally, the optimal concentration of vitamin D was recorded and analyzed.

###### Optimization of vitamin D’s optimal acting time

The CCK8 method was used to detect the anti-proliferation effect of vitamin D at multiple time points. A culture plate was taken at 12, 24, 36 and 48 hours after vitamin D stimulation, respectively, and the A450 nm value was detected. The inhibition rate of vitamin D on ASMCs proliferation at each time point was then detected, and the best time for vitamin D to act was determined.

###### HO-1 knockdown by lentiviral short hairpin RNA (shRNA)

HO-1 - targeted short hairpin RNAs were designed and synthesized by Genechem Co. Ltd (Shanghai, China). Three sh - RNA sequences (sh - RNA1, sh - RNA2, sh - RNA3) viruses (Lenti - si- HO-1), one negative control virus CON313 (hu6-MCS-CBh-gcGFP-IRES-puroomycin), two infection enhancers HiTransG A and HiTransG P were included. Following are the targeting sequences of the small hairpin RNAs: sh - RNA1: ccggagCCACACAGCACTATGTAAActcgagTTTACATAGTGCTGTGTGGcttttttg; sh - RNA2: ccgggaTGGCTTCCTTGTACCATATctcgagATATGGTACAAGGAAGCCAtctttttg; sh - RNA3: ccggacAGTGGCAGTGGGAATTTATctcgagATAAATTCCCACTGCCACTgttttttg

###### Optimization infection MOI of virus and determine the optimal infection enhancing solution

3-5×10^4^ cells/ml cell suspension was prepared in complete medium and inoculated with 2 mL into a 6-well plate. Once 20 - 30% cell fusion was reached after 24 hours, the corresponding amount of negative control virus CON313 was added according to three MOIs of 10, 50, and 100. To increase the infection efficiency, 40 ul of infection enhancing solution HiTransG A or HiTransG P or neither was added simultaneously. 72 hours later, the fluorescence was observed under the microscope, as shown in Figure S2. The results showed that the virus infection efficiency was strongest when MOI=100 and HiTransG A was present.

###### Determine the best infection efficiency of sh - RNA sequence

Based on the above conditions, ASMCs were infected with the three viruses, respectively. The fluorescence was observed by microscopy after 96 hours, as shown in Figure S3, and it could be found that the sh-RNA2 sequence had the strongest infection efficiency. Meanwhile, the knockdown efficiency of shRNA was evaluated by qRT-PCR and western blotting after 96 hours. qRT-PCR and WB showed that HO-1 was most significantly reduced in ASMCs infected with sh-RNA2 virus. Therefore, sh-RNA2 virus-infected cells was selected to constructed the stable low expression of HO-1 cell line as a follow-up experiment.

# Supplementary Figures and Tables

## Supplementary Figures

**
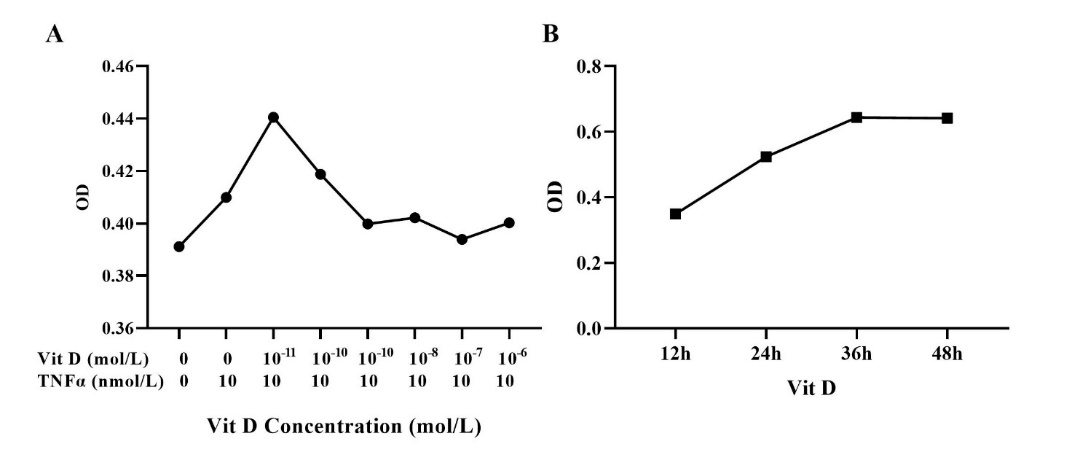
**

**Supplementary Fig. 1.** **A)** Inhibition of cellular proliferation by different concentrations of vitamin D. **B)** Inhibition of cellular proliferation by different acting times of vitamin D.

**
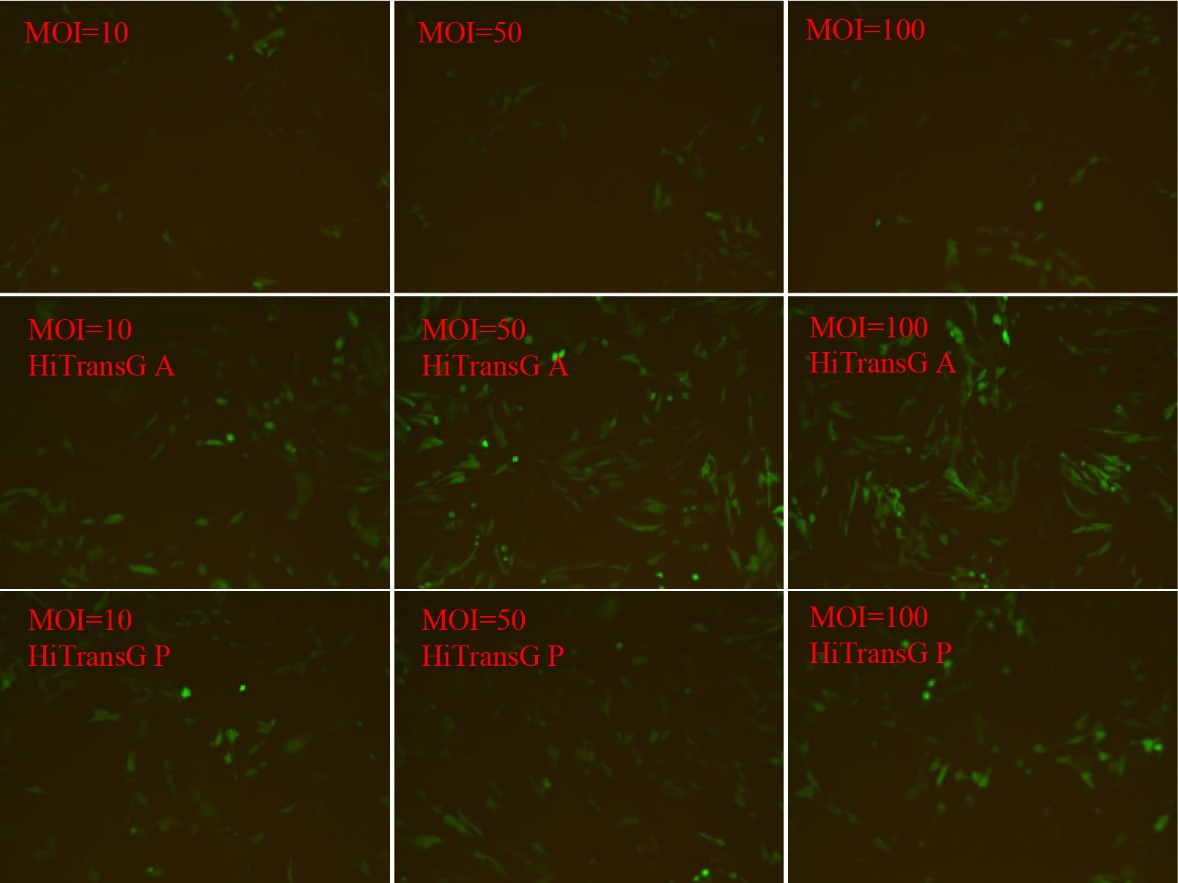
**

**Supplementary Fig. 2.** Different MOI gradients and infection enhancement solutions for cell infection efficiency

**
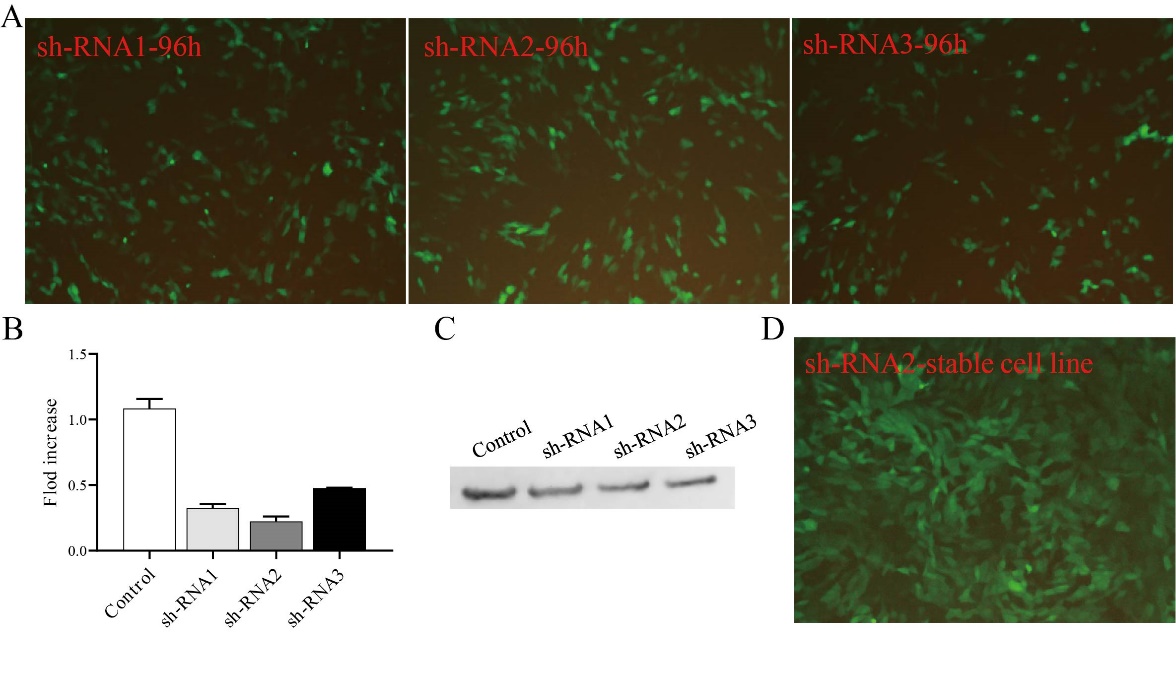
**

**Supplementary Fig. 3.** Inhibited efficiency of HO-1 sh-RNAs. **A)** ASMCs were transfected with sh-RNAs for 96 hours. **B)** HO-1 mRNA was determined by qRT-PCR. **C)** HO-1 protein expression was determined by western blot analysis. **D)** The stable low expression of HO-1 cell line of sh-RNA2 virus.

## Supplementary Table

**Table S1**. Primer sequences for qRT-PCR analyses. Primers were designed using *https://sg.idtdna.com/pages/products/custom-dna-rna* in combination with the *NCBI* and *UCSC* websites and β-actin primers from our previously published study [3]**.**

Supplementary Table 1. Primer sequences for qRT-PCR analyses.

| **Primer** | **Forward** | **Reverse** |
| --- | --- | --- |
| Nrf2 | GGACATGGAGCAAGTTTGGC | CCAGCGAGGAGATCGATGAG |
| Ho-1 | GAATCGAGCAGAACCAGCCT | CTCAGCATTCTCGGCTTGGA |
| NGF | GGAGCGCATCGAGTTTTGG | CCTCACTGCGGCCAGTATAG |
| iNOS [4] | CATGCTACTGGAGGTGGGTG | CATTGATCTCCGTGACAGCC |

## Multiple exposure images

**
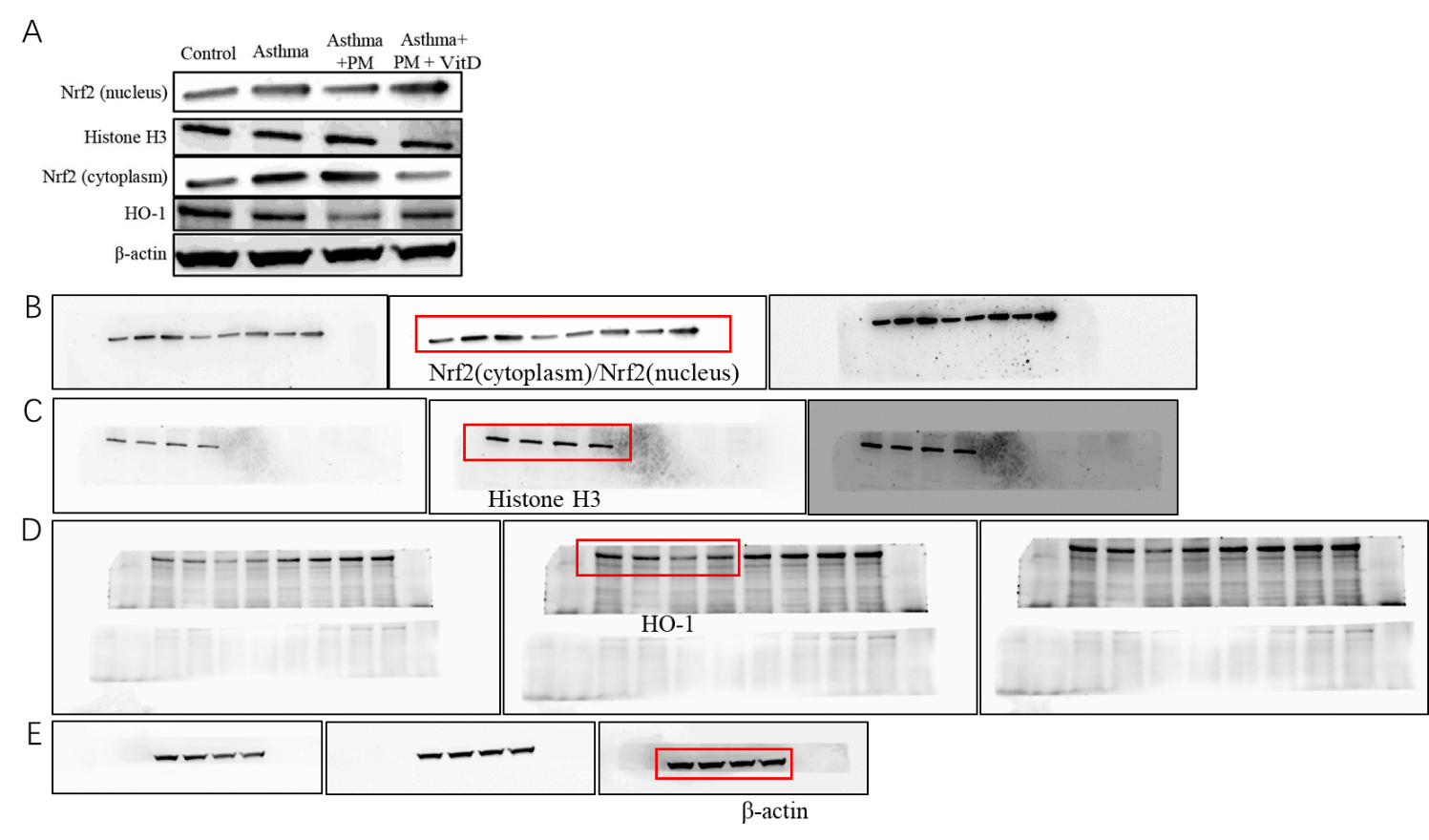
**

**Supplementary Fig.4. Full length blot for Figure 3b. A)** Immunoblot analysis of the protein expression levels of Nrf2 and HO-1 on ASMCs treated using different substances. This image is also the Figure 3b in the manuscript. **B)** The original images of cytoplasm Nrf2 and nucleus Nrf2 (Figure 3b). **C)** The original images of Histone H3 (Figure 3b). **D)** The original images of HO-1 (Figure 3b). **E)** The original images of β-actin (Figure 3b). The blots displayed in the red box represent the corresponding images in the manuscript.


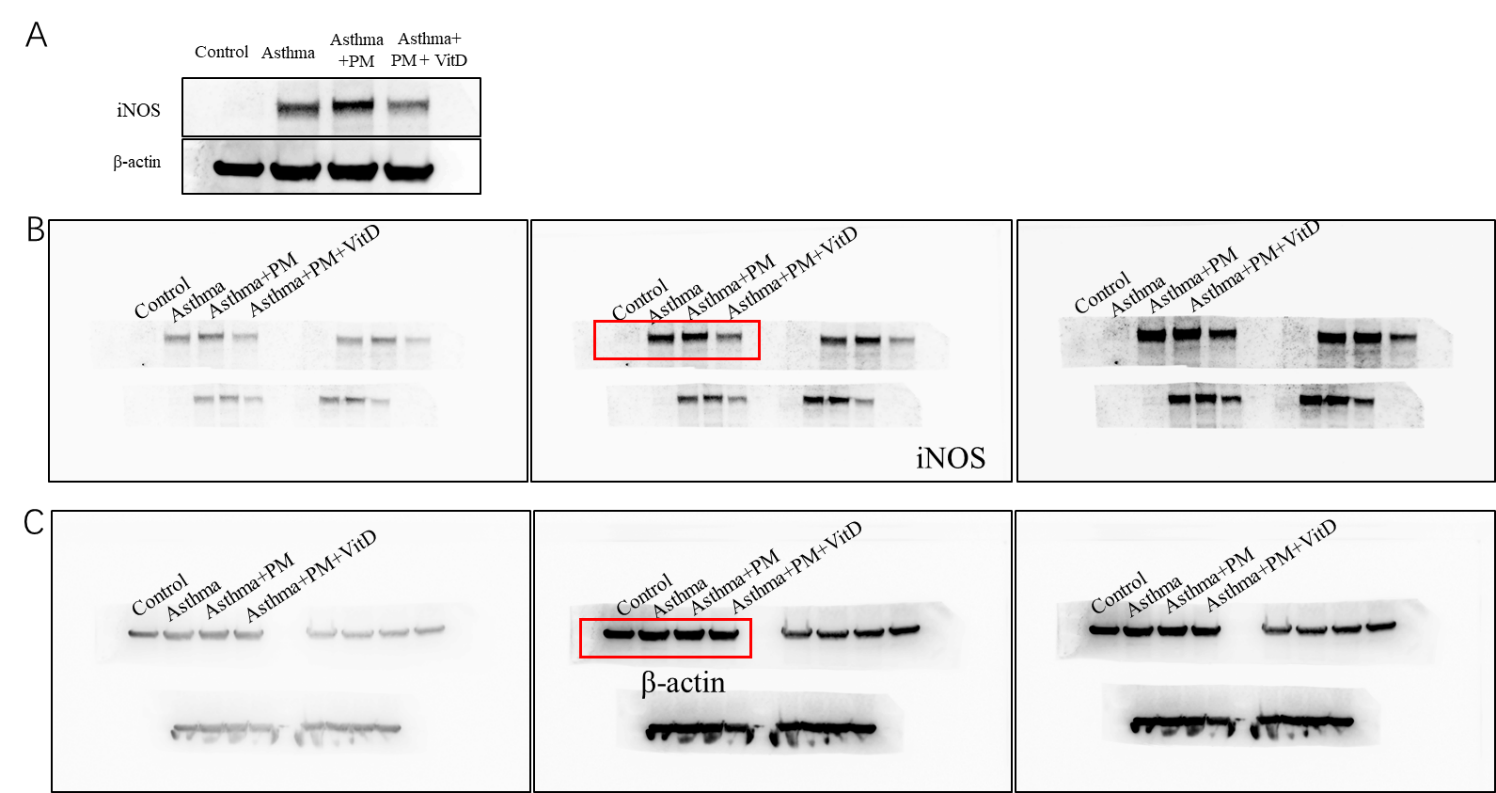


**Supplementary Fig.5.** Full length blot for Figure 3e. **A)** Immunoblot analysis of the protein expression of iNOS in lung tissue. This image is also the Figure 3e in the manuscript. **B)** The original images of iNOS (Figure 3e). C) The original images of β-actin (Figure 3e). The blots displayed in the red box represent the corresponding images in the manuscript.


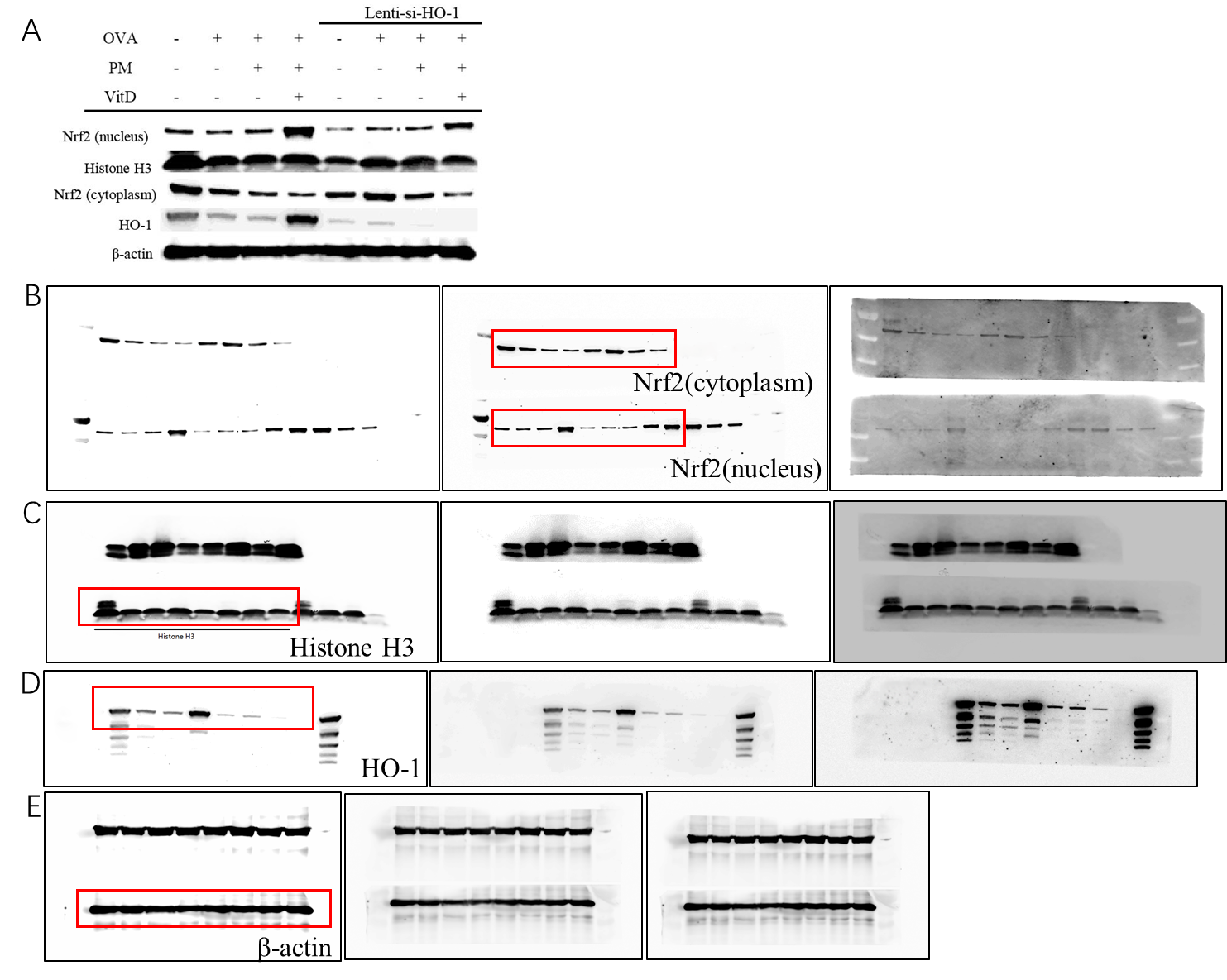


**Supplementary Fig.6.** Full length blot for Figure 4b **A)** Immunoblot analysis of the protein expression levels of Nrf2 and HO-1 on ASMCs treated using different substances. This image is also the Figure 4b in the manuscript. **B)** The original images of nucleus Nrf2 and cytoplasm Nrf2 (Figure 4b). **C)** The original images of Histone H3 (Figure 4b). **D)** The original images of HO-1 (Figure 4b). **E)** The original images of β-actin (Figure 4b). The blots displayed in the red box represent the corresponding images in the manuscript.


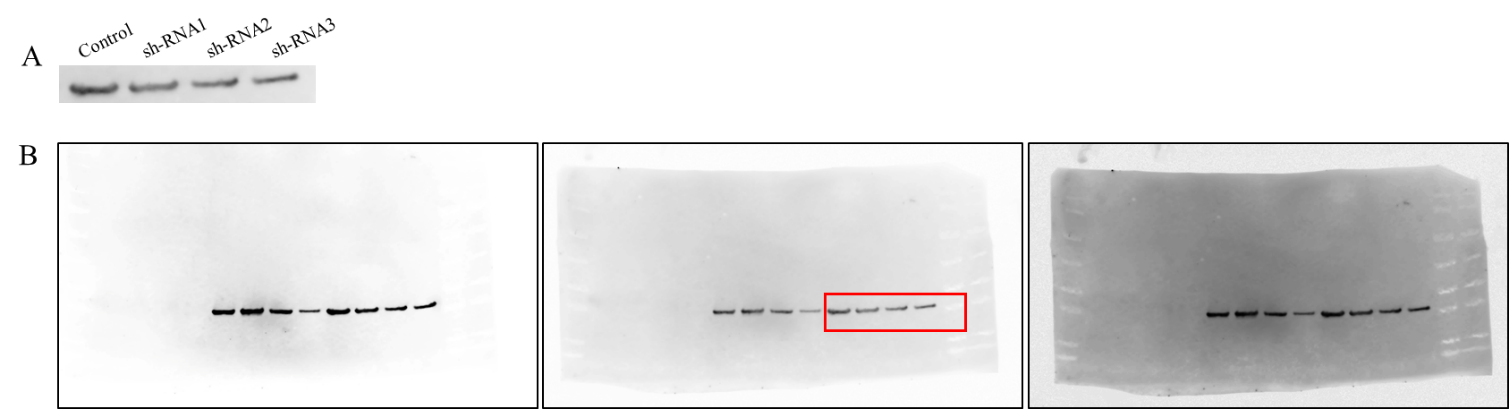


**Supplementary Fig.7.** Full length blot for supplementary figure 3C **A)** HO-1 protein expression was determined by western blot analysis. **B)** The original images of HO-1 (supplementary figure 3C).

**Reference**

1. Ford JG, Rennick D, Donaldson DD, Venkayya R, McArthur C, Hansell E, Kurup VP, Warnock M, Grünig G: **Il-13 and IFN-gamma: interactions in lung inflammation**. *Journal of immunology (Baltimore, Md : 1950)* 2001, **167**(3):1769-1777.

2. Mcmillan SJ, Xanthou G, Lloyd CM: **Manipulation of Allergen-Induced Airway Remodeling by Treatment with Anti-TGF- Antibody: Effect on the Smad Signaling Pathway**. *Journal of Immunology* 2005, **174**(9):5774-5780.

3. Wu J, Ge D, Zhong T, Chen Z, Zhou Y, Hou L, Lin X, Hong J, Liu K, Qi H *et al*: **IRF4 and STAT3 activities are associated with the imbalanced differentiation of T-cells in responses to inhalable particulate matters**. *Respir Res* 2020, **21**(1):123.

4. Trinh TA, Park J, Oh JH, Park JS, Lee D, Kim CE, Choi HS, Kim SB, Hwang GS, Koo BA *et al*: **Effect of Herbal Formulation on Immune Response Enhancement in RAW 264.7 Macrophages**. *Biomolecules* 2020, **10**(3).
